# Insulin prevents fatty acid induced increase of adipocyte size

**DOI:** 10.1080/21623945.2022.2107784

**Published:** 2022-08-22

**Authors:** Emmanuelle Berger, Alain Géloën

**Affiliations:** UMR Microbial Ecology, Research group “Bacterial Opportunistic Pathogens and Environment (BPOE)”, CNRS 5557, INRAE 1418, Lyon1 University, VetAgroSup, Villeurbanne, France

**Keywords:** Adipocyte, lipogenesis, lipid uptake, lipolysis, adiposize, realtime

## Abstract

Metabolic disorders related to obesity are largely dependent on adipose tissue hypertrophy, which involves adipocyte hypertrophy and increased adipogenesis. Adiposize is regulated by lipid accumulation as a result of increased lipogenesis (mainly lipid uptake in mature adipocytes) and reduced lipolysis. Using realtime 2D cell culture analyses of lipid uptake, we show (1) that high glucose concentration (4.5 g/L) was required to accumulate oleic acid increasing lipid droplet size until unilocularization similar to mature adipocytes in few days, (2) oleic acid reduced *Peroxisome-Proliferator Activated Receptor Gamma* (*PPARG)* gene transcription and (3) insulin counteracted oleic acid-induced increase of lipid droplet size. Although the lipolytic activity observed in high *versus* low glucose (1 g/L) conditions was not altered, insulin was found to inhibit oleic acid induced gene transcription required for lipid storage such as Cell Death Inducing DFFA Like Effectors (CIDEC) and *G0S2 (*G0 switch gene S2), possibly through PPARA activity. Although this signalling pathway requires more detailed investigation, the results point out the differential mechanisms involved in the pro-adipogenic effect of insulin in absence *versus* its protective effect on adiposity in presence of oleic acid uptake.

**Abbreviations**: AICAR, 5-Aminoimidazole-4-carboxamide-1-D-ribofuranoside; AMPK, AMP-Activated protein kinase, ASCs, adipose stem cell; ATGL, adipose triglyceride lipase; BSA, Bovine serum albumin; CEBPA, CCAAT enhancer binding protein alpha; CIDEs, Cell Death Inducing DFFA Like Effectors; dA, differentiated adipocyte; DMEM, Dulbecco’s Modified Eagle’s Medium; FABPs, Fatty Acid Binding Proteins; FAT/CD36, Fatty acid translocase; FCS, Foetal calf serum; FN1, fibronectin 1; FFA, free fatty acid; G0S2, G0 switch gene S2; GLUTs, Glucose transporters; GPR120, G protein-coupled receptor 120; HG, high glucose; HSL, hormone sensitive lipase; INSR, insulin receptor; LG, low glucose; OA, oleic acid; PBS, Phosphate buffer saline; PPARs, Peroxisome-Proliferator Activated Receptors; PKA, Protein kinase cyclic AMP-dependent; PKG, Protein kinase cyclic GMP dependent; PTGS2, cytochrome oxidase 2; RTCA, realtime cell analysis; TG, triglyceride.

## Graphical Abstract

**Figure:**
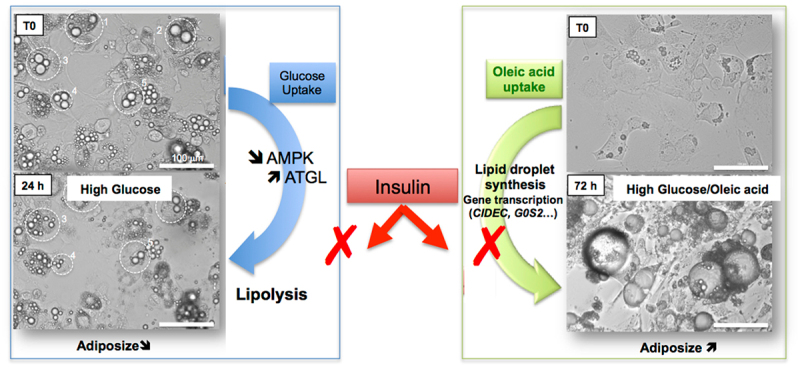

## Introduction

Metabolic disorders such as obesity or diabetes are highly dependent on adiposity due to differential capacities of fatty acid storage as well as secretion of adipokines according to adiposize [1–4]. Adipose cell size depends on long-chain fatty acid accumulation as triglycerides (TG) into a unique droplet (bimodal distribution of large cells *versus* small cells) [5–7]. In obesity, it results from additional (1) adipose cell hypertrophy (increased population of large cells *versus* small cells) as a result of TG accumulation and (2) hyperplasia due to increased adipogenesis and increased proliferation of adipose stem cells (ASC). Adiposize increase is a result of both increased TG storage regulated by transcriptional regulation of genes involved in fat storage (increased) and fat utilization (reduced) [8]. Fat storage in adipocytes results from several mechanisms including: (1) free fatty acid (FFA) uptake and storage as TG into droplets *versus* FFA release (lipolysis) mainly hormone-sensitive dependent [1,9]; (2) Intracellular signalling through binding to specific receptors (e.g. fatty acid tanslocase FAT/CD36, G protein-coupled receptors including GPR120), interaction to intracellular fatty acid binding proteins (FABPs) leading to protein modifications and/or addressing of transporters (Glucose transporter GLUT4) and receptors (FAT/CD36) as well as (3) nuclear translocations leading to transcriptional regulations involved in lipogenesis and adipogenesis (e.g. Sterol regulatory element binding proteins SREBPs, peroxisome proliferator activated receptor PPARs); (4) mitochondrial fatty acid *de novo* synthesis from glucose at least in immature cells [1,10–13]. Adiposize finally results from cumulative effects of lipid uptake, lipogenesis and lipolytic mechanisms differentially controlled during time by glucose oxidation (1 h); glucose uptake (1h30), through glucose transporters GLUT1/GLUT4 (2–4 h) [14–19] *versus* protein and transcriptional regulations (up to several hours) [20].

The study of adiposize regulation requires *in vitro* approaches. In isolated adipocytes inflammatory responses are increased [7], their lifetime is reduced and depends on gender, age, depot or sex specific differences [21]. Therefore, we recently developed an experimental strategy using human adipose stem cells (ASCs) in order to study adipogenesis and lipogenesis in 2D cell cultures, in similar conditions as 3T3L1 fibroblast cell line, in realtime experiments using xCELLigence system (cell number, cell size, cell adhesion force parameters) combined with realtime imaging and TG storage quantifications by fluorescence [22,23]. The formation of unilocularized adipocytes required treatment with fatty acids such as oleate, a fatty acid which in excess has previously been found to influence adipogenesis by increasing PPARG and CEBPA activation through DNA methylation, thus increasing predisposition to obesity at least *in vitro* [24]. We also observed that although insulin increased lipid content in differentiating adipocytes (dA), lipid accumulation was reduced in adipocytes treated by oleic acid, suggesting a positive effect of insulin on lipogenesis under low fatty acid extracellular content during adipogenesis, but protection against high fat-induced TG accumulation [22]. The aim of the present study was to validate such an hypothesis and to decipher the signalling pathways involved in the regulation of adiposize.

Lipid accumulation results from several mechanisms including *de novo* lipid synthesis, lipid uptake as well as lipolysis. At a given time, intracellular lipid, that is, size and number of lipid droplets, can be considered as the result of the balance between lipogenesis and lipolysis [23]. The resulting accumulation of TG is heterogeneous, depending on differentiating state as well as cell culture heterogeneity, with better linearity in 3T3-MBX (sub-clone 3T3L1 cell line with differentiation efficiency >90%) than in 3T3L1 cell line. In previous studies, we observed an improved intracellular lipid accumulation during adipogenesis in response to oleic acid than with high glucose alone [22]. Nevertheless, since free fatty acids are toxic, their concentration must be optimized to reduce their toxicity on poorly differentiated cells but high enough to increase lipid droplet formation. For this purpose, we optimized the method to analyse adiposize restricted to droplets with at least 50 µm diameter by comparison to adipocytes from rat epididymal explants. The present study shows that (1) in high glucose media, basal lipolysis occurs, (2) high glucose concentration is required to optimize induction of adiposize increase by oleate and (3) adiposize increase due to oleic acid uptake, is (4) reduced by the counteracting effect of insulin on gene transcription coding for proteins involved in lipid storage.

## Results

### 1- Oleic acid promotes adipocyte maturation with inhibitory effect of insulin.

**Figure 1.**
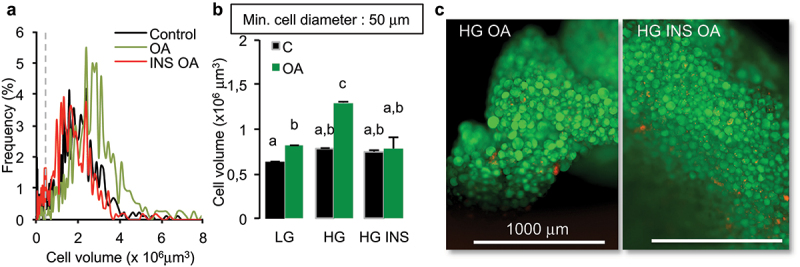
Insulin counteracts oleic acid induced increase of adiposize in rat epididymal explants.

In order to study the regulation of adiposize by fatty acids, oleic acid, glucose and insulin were used in combination at optimized concentrations (i.e. 10 µM, 4.5 g/L, 0.05 U/mL), respectively). Rat epididymal adipose tissue explants were treated during 48 h. Mature adipocyte diameters are in a range of 50 µm^7^. Oleic acid (10 µM) induced an increase of adiposize which was higher in high glucose containing media (4.5 g/L, HG) *versus* low glucose (1 g/L, LG) media, almost a doubling in mean cell volume was observed in HG media after 2 days (Figure 1). This effect was abolished by insulin (0.05 U/mL).

Explants of approx. 1 mm^3^ were treated during 48 h in culture media with oleic acid (OA 10 µM) in either 1 g/l (LG), or 4.5 g/L (HG) with or without insulin (INS 0.05 U/mL), then fixed and labelled with AdipoRed for quantification on x4 images. (A) Adiposize distribution of representative samples. (B) Mean adipocyte volumes ± SEM with significant differences represented by different letters (Anova tests, 3 biological replicates, 10 fragments per replicate). (C) Representative merged images of explants labelled with AdipoRed (green) and Hoechst (red).

**Figure 2.**
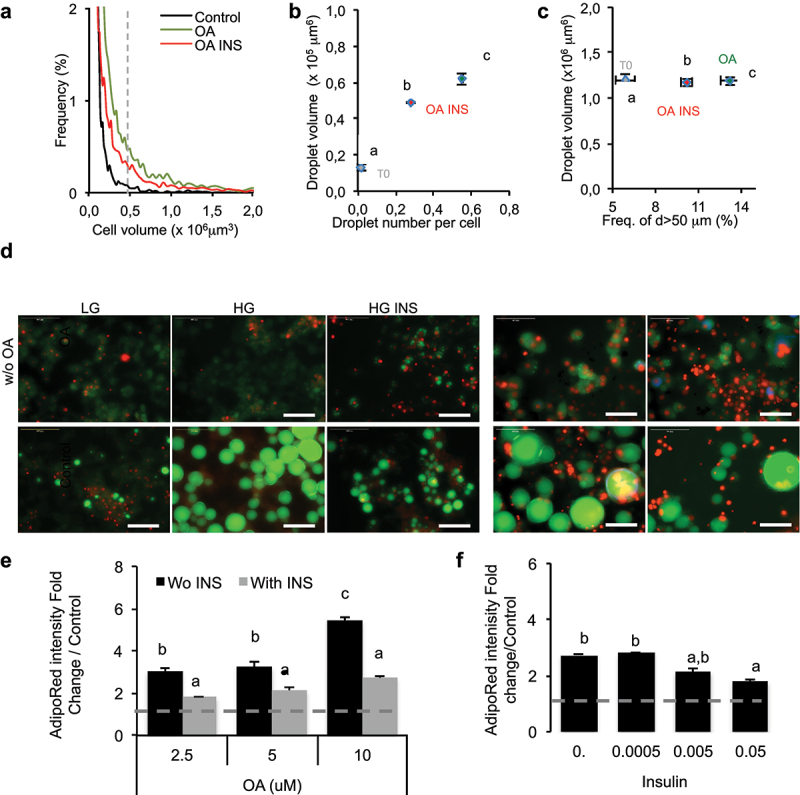
Oleic acid uptake increases adiposize with inhibitory effect of insulin.

In order to study the mechanisms involved in the regulation of adiposize, a comparative analysis of oleic acid treatment of 3T3L1 and its subclone 3T3-MBX adipocytes was performed after optimization of cell culture conditions (Figure S1). By comparison to adipose cells, in cell cultures, mature adipocytes size frequency was considered from either cell or droplet size up to 50 µm, that is, in a volumic range up to 0.6 x 10^6^ µm^3^. During adipogenesis, oleic acid induced significant increase of adipose cell size in 3T3L1 cells (Figure 2(a)), droplet volume and number (Figures 2(b)) and the frequency of droplets with up to 50 µm diameter (Figure 2(c,d)). The effect of insulin used at the concentration required for differentiation (0.05 U/mL) was dependent of the dose of oleic acid up to 10 µM (Figure 2(e)), inversely the effect of OA at 10 µM was reduced in a dose-dependent manner by insulin up to 0.05 U/mL (figure 2(f)).

(A) Adipose cell size analyses of 3T3L1 adipocytes treated during 3 days on Multisizer cell counter (10 000 cells/condition, n = 4 biological replicates), (B) by droplet volume and number per cell and (C) frequency of droplets of at least 50 µm diameter by image automated cell quantification (n = 8 biological replicates). (D) Representative merged AdipoRed (green) and Hoechst 33,258 (red) images at objective x20 of partially differentiated 3T3L1 adipocytes treated during either 24 h or 3 days with LG (1 g/L) or HG (4.5 g/L) with or without INS (0.05 U/mL) or OA (10 µM).E-F Lipid content in 3T3-MBX adipocytes treated during 3 days with (A) several doses of oleic acid in HG with or without INS (0.05 U/mL) or (B) several doses of INS with OA 10 µM. Different letters represent significant differences (p < 0.05, Anova tests).

In fact, as previously observed in both 3T3-L1 and human ASCs [22], although adipogenesis induction by insulin requires long-term cell culture (more than 2 weeks), only 24 h–3 days treatment with oleate after adipogenesis induction are required to obtain fully differentiated adipocytes. Insulin significantly reduced oleic acid induced adipogenesis although it was not completely abolished, compared to control. In 2D cell cultures as well as on explants, adipocytes tend to detach when treated with high doses of oleate during 24 hr (not shown), thus suggesting requirement of additional extracellular matrix synthesis for cell attachment. Realtime imaging of oleic acid uptake by 3T3-MBX dA during 24 hr (S2A videos) and contrast phase images after 4 days revealed that although homogeneous differentiation, adiposize increase was highly heterogeneous. Another important parameter allowing unilocularization and hypertrophy was the dose of oleic acid applied, which depends on the number of differentiated cells and the size of lipid droplets. Thus, the mean dose of fatty acid applied must be a compromise between toxicity of poorly differentiated cultures, the risk of cell detachment of hypertrophic cells and the quantity of fatty acid required for unilocularization and hypertrophy. In order to standardize all the experiments and avoid cell detachment, the treatments were performed with OA 10 µM during 4 hr to 3 days.

### 2- Insulin promotes increase of lipid droplet size through inhibition of lipolysis induced by high glucose.

Adiposize results from additional effects of lipid uptake plus synthesis (lipogenesis), degradation and release (lipolysis). Both lipid uptake and release are short-term events measured during several hours. In RTCA (Realtime cell analysis) experiments oleate uptake can be detected from few minutes to several hours depending on dose and differentiation state [22].

**Figure 3.**
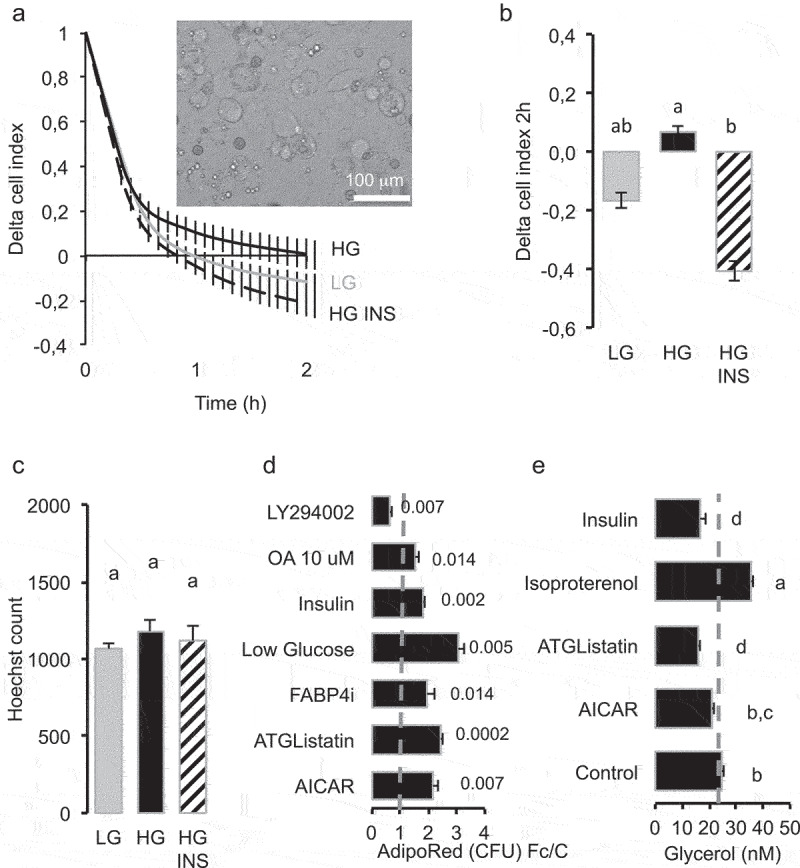
Basal lipolysis induced by high glucose (4.5 g/L) during 2 hours is prevented by insulin in 3T3L1 partially differentiated adipocytes.

**Figure 4.**
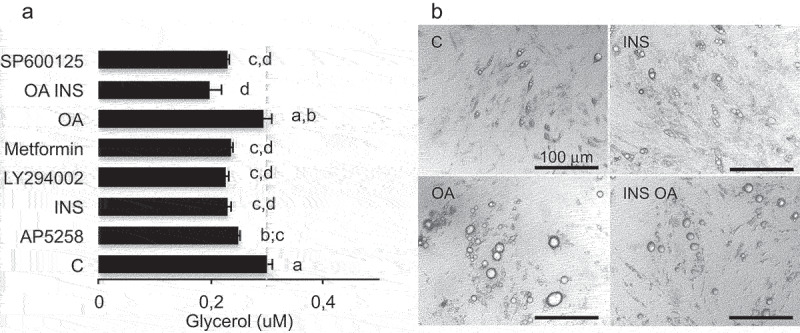
Insulin prevents basal lipolysis induction by high glucose in human adipocytes independently of oleic acid.

During 2 hours of culture, we observed that cell index decreased with time in partially differentiated adipocytes independently of culture media. This suggests that the *de novo* lipogenesis process was not altered (Figure 3(a)). However, the cell adhesion force was less reduced in HG media by comparison to LG media after 2 hours, in accordance with a lower TG content in cells maintained in HG *versus* LG media (Figure 3(b)) and without significant toxicity (Figure 3(c)). Lipid content in short-term experiments (4 h) was monitored in 3T3-L1 adipocytes and the lower accumulation in HG was reversed by insulin, inhibition of either FABP4 or Adipocyte triglyceride lipase (ATGL) or by activation of AMP-Activated protein kinase (AMPK), although the inhibitory effect of insulin was reversed by inhibition of PI3 kinase (Figure 3(d)). The quantification of free glycerol release confirmed the role of a lipolytic activity of glucose inhibited by insulin together with the results obtained with either ATGL inhibition or AMPK activation (Figure 3(e)). For longer exposure times, that is, 48 h in HG culture media, lipolysis was also detected in human adipocytes, with an inhibitory effect of insulin (Figure 4). However, in these times of exposure, the signalling pathways regulating glycerol release were different, reduced by LKB1/AMPK activation by metformin, reduced when FAT/CD36 and PI3kinase were inhibited.

(A) Realtime analysis of cell adhesion on xCElligence system, represented by delta cell index (normalization at time of treatment) according to time in low glucose (LG, 1 g/L) *versus* high glucose (HG 4.5 g/L) and HG with insulin (INS 0.05 U/mL). (B) Mean delta cell indexes 2 hours after treatment (Fisher test). (C) Cell number by Hoechst 33,258 labelled nuclei counts on x4 images. (D) Effect of insulin and inhibition of lipolysis on (TG) content in 3T3-L1 adipocytes, differentiated in HG plus INS media then treated during 4 h with LG, or HG with INS, Pi3 kinase inhibitor LY294002 (10 µM), oleic acid OA (5 µM), lipolysis inhibitors: AMPK activator AICAR (1 mM), ATGL inhibitor ATGListatin (1 µM), FABP4 inhibitor FABP4i (20 µM). TG content was measured as the fold change of AdipoRed fluorescence intensity to that of control media (HG, no significant effect of inhibitor solubilization media, not shown). Only significant pathways are presented (Student’s test p-values<0.05, n = 8 biological replicates), full data are provided in Table S2. The results were obtained from independent experiments for each treatment.E- Regulation of lipolysis assessed by quantification of free glycerol release during 2 hours. Induction of lipolysis by high glucose in DMEM without serum was quantified in the presence of either human recombinant insulin 0.05 U/mL, isoproterenol (100 nM) as positive control, ATGListatin and AICAR with the same concentrations as in (D). In B, C and E, data are presented as mean values ± SEM (n = 8 biological replicates), different letters represent significant differences (Anova variance tests).

(A) Glycerol release by human *in vitro* differentiated adipocytes treated during 48 h in HG culture media with serum. Data are presented as mean values ±SEM (n = 8 biological replicates) with letters representative of significant differences (Anova variance test (p < 0.05). Full data are provided in Table.S3 (B) Phase contrast images of human adipocytes.

**Figure 5.**
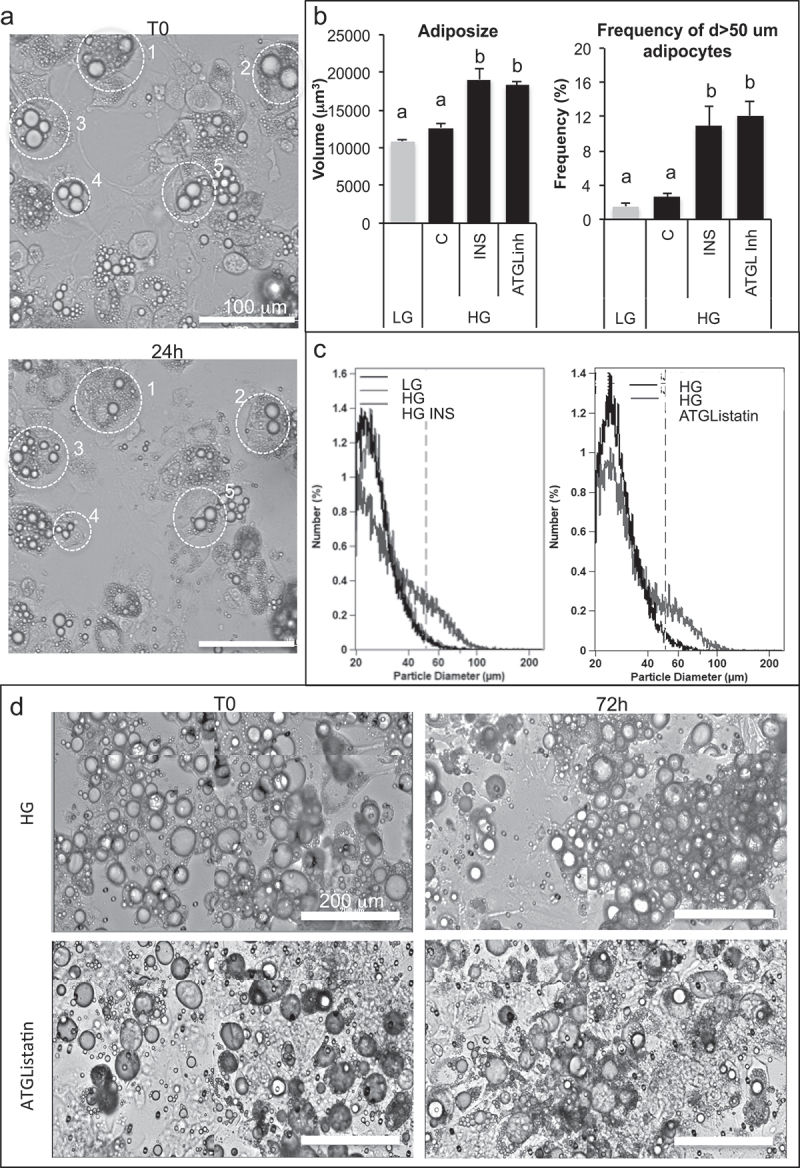
Anti-lipolytic effect of insulin results in an increase of adiposize.

In realtime experiments, high glucose induction of lipolysis in 3T3L1 adipocytes was observed independently of adiposize (Figure 5(a)). Adipogenesis and adiposize increase due to fatty acid accumulation was highly heterogeneous, allowing to obtain large unilocular droplets. Small lipid droplet size reduction *versus* larger lipid droplets size increases were observed (S2B Videos), thus suggesting a major role of basal lipolysis in small droplet-containing adipocytes, and an increased lipid uptake capacity according to adiposize.

(A) Realtime imaging of basal lipolysis induced by high glucose in 3T3L1 adipocytes: examples of cell size reduction are shown by circled examples 1–5 at T0 and 24 h later. (B–D) After treatment during 3 days with oleic acid 10 µM, fully differentiated 3T3-MBX adipocytes were maintained during 3 days in culture media with either low glucose (LG, 1 g/L) or high glucose (HG 4.5 g/L), insulin (INS 0.05 U/mL) or lipolysis inhibitor ATGListatin then cell size distribution was analysed on Multisizer (10 000 cells). (B) Full cell sample size distribution (left panel) and size distribution of cells with diameter up to 50 µm (right panel). Data are presented as mean cell counts ±SD with significativity represented by letters for multivariate Fisher tests (n = 4). (C) Cell size distribution histograms. (D) Contrast phase images of cells monitored in realtime from T0 to 72 h (scale bars = 200 µm).

In order to compare the results obtained with partially differentiated 3T3L1 adipocytes, 3T3-MBX adipocytes were pre-treated with oleic acid during 3 days before testing whether basal lipolysis occurs (Figures 5(b)). Cell size distribution analyses revealed that both insulin and ATGL inhibition increased adiposize, thus confirming that inhibition of basal lipolysis is required to improve adiposize increase.

For more convenience, we propose to use the term of basal lipolysis induced by glucose by distinction with lipolysis induced by catecholamine (such as isoproterenol), in the next experiments. Noticeably, in short term response analyses (2–4 h) it was possible to measure lipolysis, although after longer exposure times (24 h–3d, i.e. Figure S1) the increase of adiposize revealed an upper ratio of *de novo* lipid synthesis *versus* basal lipolysis. Taken together, these results indicate that insulin promotes adipogenesis by counteracting HG-promoted basal lipolysis through PI3 kinase signalling pathways, although AMPK reduces basal lipolysis in differentiating adipocytes. Moreover, during fatty acid induced increase of adipocytes, the protective effect of insulin involved different cellular mechanisms from direct regulation of lipolysis. Therefore, its activity on transcriptional regulations by oleic acid was further explored.

### 3- Insulin prevents oleic acid induced increase of lipid droplet size through down regulation of adipogenic gene transcription

**Figure 6.**
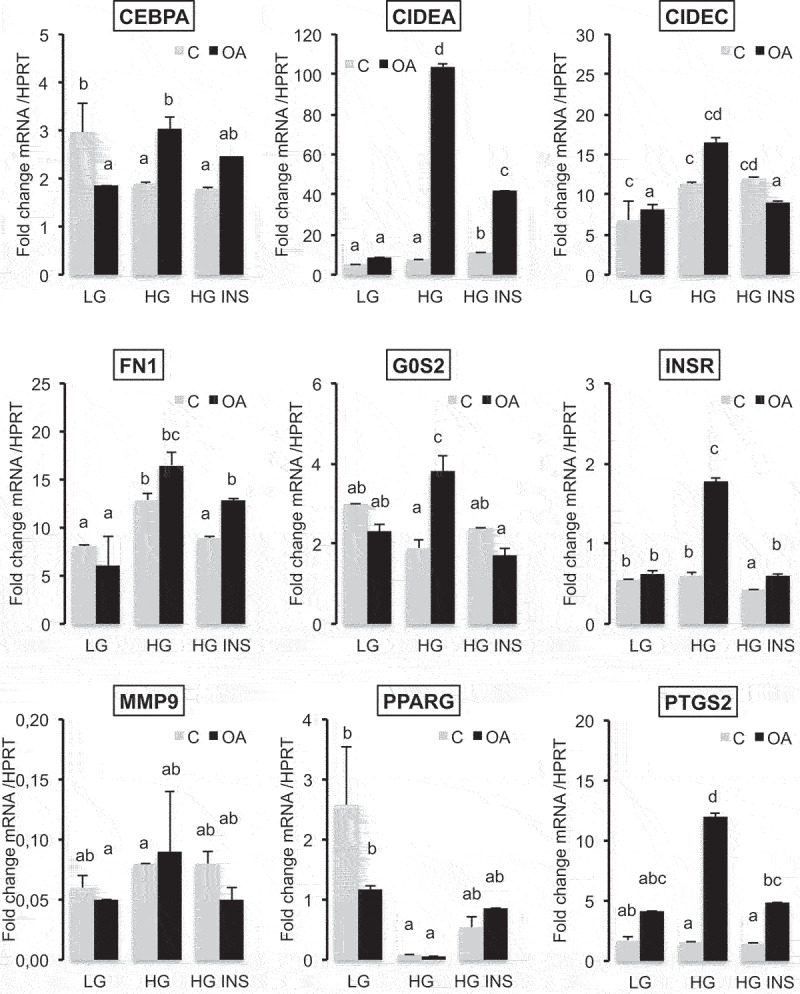
Oleic acid-induced gene transcription of adipogenic markers in high glucose media is counteracted by insulin in 3T3L1 adipocytes.

A selection of genes involved in adipogenesis and/or lipid metabolism have been selected to further analyse the transcriptional regulations in 3T3L1 dA in either LG, HG or HG with insulin with or without OA (24 h, Figure 6). In comparison to LG, only fibronectin *FN1* and *CIDEC* were significantly induced by HG. Insulin counteracted the effects of HG in reducing *FN1* and reduced both *PPARG* and its own receptor (*INSR*) gene transcription.

qRT-PCR analysis of genes 24 h after treatment with either low glucose (LG 1 g/L), high glucose (HG 4.5 g/L) in the presence of insulin (INS 0.05 U/mL) and/or oleic acid (OA 10 µM) culture media with serum. Data are presented as mean relative concentration values ±SD normalized to that of HPRT (hypoxanthine phosphoribosyltransferase). *CEBPA*, CCAAT enhancer binding protein alpha; *CIDEA* and *C*, cell death inducing DFFA like effectors A and C; *PTGS2* (*COX2)*, cytochrome c oxidase subunit II; *FN1*, fibronectin 1; *G0S2*, G0/G1 switch 2; *INSR*, insulin receptor; *MMP9*, matrix metallopeptidase 9; *PPARG*, peroxisome proliferator activated receptor gamma. Significant differences are represented by different letters according (Anova variance analyses, n = 3 biological replicates, 4 technical replicates).

In the presence of OA, the transcriptional responses were highly increased in HG *versus* LG media. *CEBPA* (CCAAT enhancer binding protein alpha), *CIDEA, CIDEC, FN1, G0S2 (*G0 switch gene S2), *INSR, MMP9* (metalloprotease 9) and *PTGS2 (*cyclooxygenese 2) were highly induced by OA in HG media. Only *PTGS2* was slightly induced by OA in LG media. *PPARG* gene transcription was not modulated. In HG with OA, insulin exerted an inhibitory effect on OA mediated gene transcription for all these genes except *FN1, MMP9 and PPARG*.

**Figure 7.**
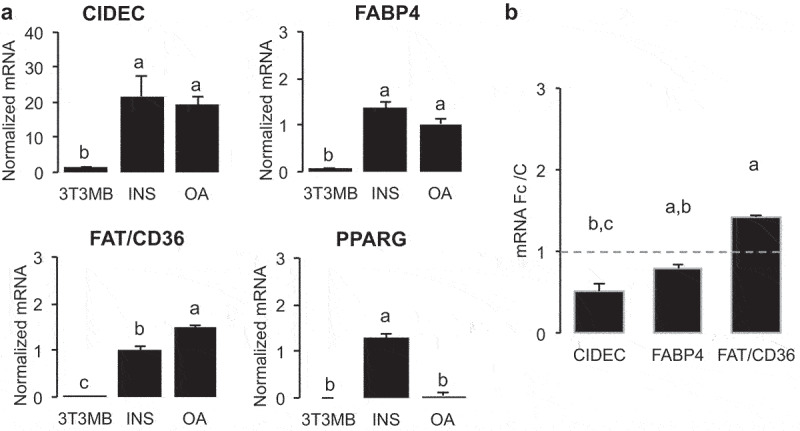
Oleic acid increases adipogenic markers in 3T3-MBX adipocytes with repressive effect of insulin.

**Figure 8.**
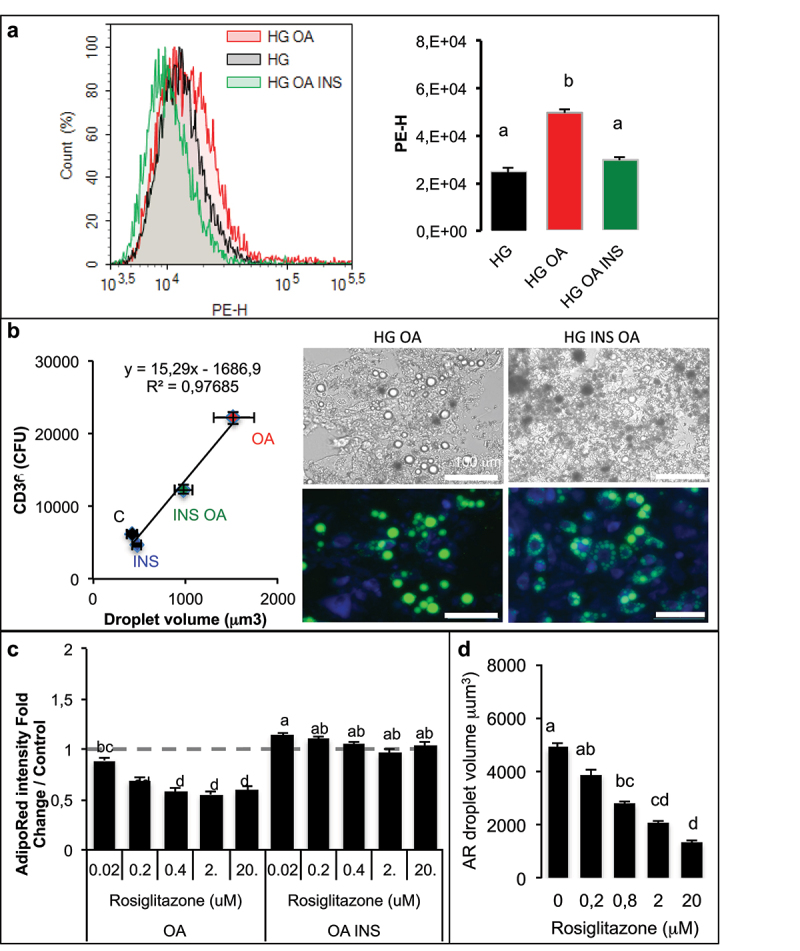
Although oleic acid increases adipogenic marker FAT/CD36 with repressive effect of insulin, PPARG activation reduces OA-induced lipid accumulation in adipocytes.

In 3T3-MBX adipocytes (Figure 7(a)), both insulin and OA highly induced the adipogenic markers *CIDEC, FABP4* and *FAT/CD36* in HG culture media. However, although *PPARG* was induced by insulin, it was repressed in the presence of oleic acid. Similar results were obtained in human adipocytes (Table S4, Figure S5). In adipocytes pre-treated with OA in order to increase adiposize (Figure 7(b)), insulin increased *FAT/CD36* gene transcription, although that of *CIDEC* was repressed. In differentiated 3T3-MBX adipocytes FAT/CD36 expression was confirmed using fluorescently labelled anti-FAT/CD36 antibodies directed against its extracellular domain (Figure 8). Its induction by oleic acid was counteracted by insulin.

(A) Gene transcription analysis in 3T3-MBX proliferating cells (3T3MB), 2 days post-differentiated 3T3-MBX cells maintained in insulin-containing culture media (INS 0.05 U/mL) or treated during 3 days with oleic acid 10 µM (OA).B- Gene transcription analyses in 3T3-MBX adipocytes treated during 24 h with HG OA 5 µM then with insulin 0.05 U/mL during 24 h. Data were normalized from time of treatment with insulin.In (A) and (B) mRNA concentrations obtained from delta Ct were normalized to those of HPRT. Data are presented as mean normalized mRNA ±SD (n = 4 biological samples and 4 technical replicates) with different letter for significant differences (Fisher test).

3T3-MBX adipocytes were treated during 3 days in high glucose culture media (HG) with OA (10 µM) with or without insulin (INS 0.05 U/mL) then labelled without permeablization with a phycoerythrin (PE-H) labelled antibody directed against the extracellular domain of FAT/CD36. PE was quantified by cell sorting (A) and lipid droplet size measured after AdipoRed counterstain on x4 images (B). Data are presented as mean values ±SD with letters representative of significant differences (Anova test, n = 8 biological replicates and 4 technical replicates) (A) and linear tendance curve with significant correlation coefficient (B). (B) Representative images at x20 in phase contrast and merged PE-antiCD36 (blue) and AdipoRed (green). (C) Dose-dependent response to rosiglitazone (µM) measured by quantification of fluorescence incorporation of AdipoRed presented as fold change Adipored intensity in adipocytes treated in OA or in OA with INS culture media and (D) lipid droplet size on images x4. Different letters represent significant differences (Anova and Tukey tests on 8 replicates).

The unexpected repression of PPARG gene transcription by oleate (Figure 7(a)) suggests its implication in the regulation of adiposize. In a dose-dependent analysis of rosiglitazone effect, the lipid accumulation due to oleic acid uptake was reduced after 3 days, although PPARG activation did not affect OA-induced TG accumulation in presence of insulin (Figure 8(c)). The inhibitory effect of rosiglitazone on TG accumulation induced by OA was related to reduction of droplet size (Figure 8(d)).

### 4- Signalling pathways involved in lipid droplet size regulation by glucose, insulin and fatty acids

**Figure 9.**
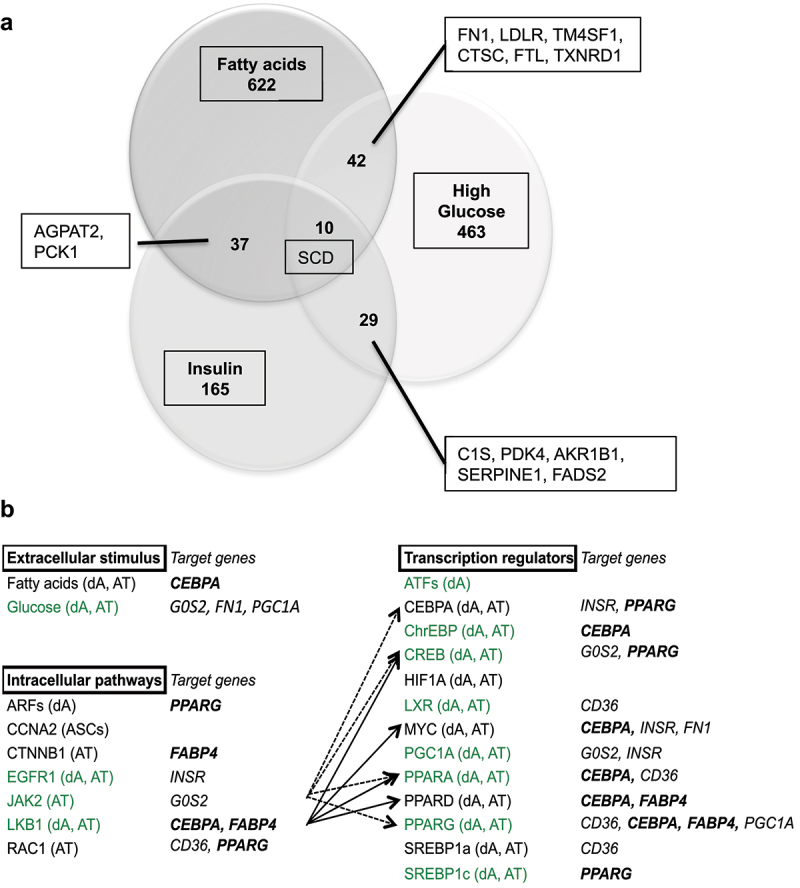
Gene dataset analyses of common transcriptional regulations by glucose, insulin and fatty acids in human cells (a) and in adipose tissue (b).

The transcriptional pathways commonly regulated by glucose, insulin and fatty acids in human cells were analysed using gene dataset comparisons (Figure 9) as previously described [22,25], showing that 42 genes are commonly regulatable by fatty acids and HG, 37 by fatty acids and insulin, 29 by insulin and HG. One gene involved in fatty acid synthesis, *SCD*, is commonly regulatable by the 3 extracellular signals. Among the genes regulatable by at least 2 of the signals, several are involved in the regulation of fat mass and obesity, that is, in either lipid metabolism (AGPAT2, FADS2, LDLR, PDK4, SCD and TXNRD1), extracellular matrix (FN1, SERPINE1), stress and/or inflammation (AKR1B1, C1S, CTSC) (Table S6).

(A) Genes commonly regulatable by glucose, insulin and fatty acids. Genes related to regulation of fat mass and obesity are reported (see Table S6 for gene names and function). (B) Insulin signalling pathways commonly regulatable by fatty acids in human adipose tissue gene datasets. Gene datasets were retrieved from the literature (Table S7A), first submitted to individual analysis of significant enrichment for glucose [25], insulin, fatty acids, and adipose tissue represented by AT (adipose tissue), dA (*in vitro* differentiated adipocytes) and ASCs (adipose stem cells) (Figures S7 B–F). Datasets regulatable by glucose are green typed, adipogenic markers commonly identified in mouse cell lines and human adipocyte lineage (Table S4) are in bold italic characters. Significativity was determined for z-test confidence level >95%.

We have previously described the signalling pathways regulatable by glucose [25] using comparative analyses of human gene datasets. This method was applied in order to describe the signalling pathways (i.e. extracellular stimulus, intracellular pathways and transcriptional regulators) specifically regulatable by insulin and fatty acids in adipose tissue (Table S7A for gene dataset references, Figures S7B–D for signalling pathway enrichments). Insulin and fatty acids commonly regulate the activity of adipogenic factors CEBPA, SREBP1 and PPARs, together with the glucose responsive factor ChrEBP. CEBPA is also the transcriptional activator of *PPARG*. The three extracellular signals may commonly regulate gene transcription through Epidermal Growth Factor Receptor (EGFR), Janus kinase 2 (JAK2) and LKB1 signalling. Interestingly, insulin and fatty acids are commonly enriched in 13 transcriptional regulators target genes, 8 of them are also regulatable by glucose, including ChrEBP and PPARs.

A panel of selective activators and/or inhibitors, including those of LKB1 and JAK2 were used at optimized concentrations (Table S8) in order to explore the role of the candidate signalling pathways merged from this study. Lipid droplet sizes were analysed in both 3T3-MBX and 3T3L1 adipocytes treated during 3 days with oleic acid in the presence of insulin (Table 1, Table S4). Only PPARA inhibition induced an increase of droplet size in both 3T3L1 and 3T3-MBX adipocytes, although that of PPARG was significantly efficient only in 3T3L1 adipocytes. These results confirm that insulin regulates adiposize through signalling pathways regulating gene transcription.

**Table 1.** Effect of insulin (0.05 U/mL) on lipid droplet volumes in adipocytes treated in presence of oleic acid (10 µM). Data are presented mean fold changes of droplet volume in samples treated during 3 days with selected drugs to corresponding control HG + INS + OA media (Fc/C) ±SEM. Data were obtained in independent experiments; significant Student t-test p-values p < 0.05 are indicated in italics (n = 8 biological replicates, at least 2 images per sample) .

		3T3-MBX				3T3L1			
Drug	Target	Fc/C		sd	*p-value*	Fc/C		sd	*p-value*
AP5258	FAT/CD36 inhibitor	1.16	±	0.25		1.53	±	0.25	
ATGListatin	ATGL inhibitor	0.81	±	0.03		1.49	±	0.11	
GSK13764747	GPR120 activator	0.94	±	0.08		0.45	±	0.03	*0.027*
GW6742	PPARA inhibitor	1.60	±	0.08	*0.002*	1.73	±	0.15	*0.002*
GW9662	PPARG inhibitor	1.34	±	0.07		3.44	±	0.50	*0.017*
KN93	Cam Kinase II inhibitor	0.96	±	0.05		1.39	±	0.12	*0.022*
LY20094	Pi3 kinase inhibitor	1.23	±	0.06		1.4	±	0.11	
Metformin	LKB1/AMPK activator	1.11	±	0.08		1.69	±	0.10	*0.004*
Tyrphostin AG-490	JAK2 inhibitor	0.90	±	0.04		0.79	±	0.07	
U73122	PKC inhibitor	0.59	±	0.03	*0.003*	0.85	±	0.10	
WZ4003 20 uM	NUAK1 inhibitor	0.89	±	0.04		0.8	±	0.06	
WZ4003 100 uM	NUAK2 inhibitor	0.86	±	0.05		1.74	±	0.24	

Taken together, these results show that *in vitro* adipogenesis is increased by insulin, although oleic acid induction of adiposize increase is repressed by insulin.

## Discussion

Adipose cell cultures in 2D have been extensively used to study the regulation of adipose cell differentiation, lipogenesis and lipolysis. Nevertheless in the vast majority of the studies, cells are considered to be adipocytes as soon as they content lipid droplets whatever are the size and number. In most of the studies, adipose cells are multilocular, that is, containing many small lipid droplets, while *in vivo* adipocytes contain a unique large lipid droplet (unilocular). To mimic at the best *in vivo* conditions in a 2D cell culture model, it is of importance to obtain high content lipid adipose cells, ideally unilocular. Present results show that high glucose and the presence of insulin markedly increase lipid content of 3T3-L1 cells (Figure S1) and significant increase of size and number of lipid droplets were observed only after 3 days. Another way to improve intracellular lipid deposition was to add fatty acid in the culture medium. It strongly and rapidly increased lipid droplet volume and the frequency of adipose cells bigger than 50 µm (Figure 2). In such conditions, insulin was found to reduce lipid content in rat adipose tissue explants, 3T3L1 and 3T3-MBX adipocytes exposed to OA. This inhibitory effect of insulin was also observed in 3T3L1 and 3T3-MBX treated with palmitic acid (not shown).

**Figure 10.**
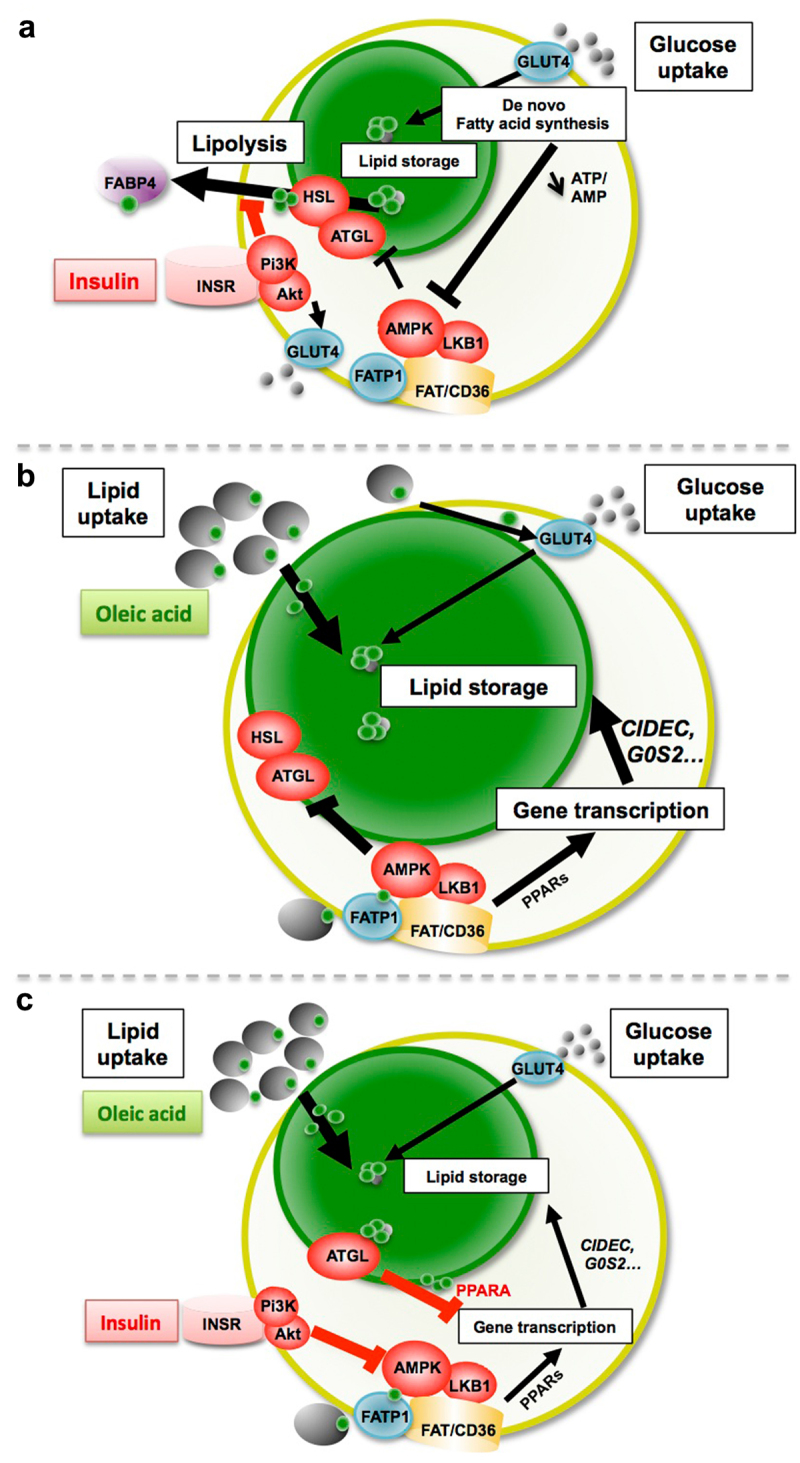
Hypotheses describing the main pathways regulating adiposize.

The regulation of adiposize is a result of (1) short-term events including lipid uptake, *de novo* fatty acid synthesis, lipolysis and (2) longer time-consuming events, that is, transcriptional regulations and synthesis of the protein machinery (required for structural adaptation to several ten fold volume increase upon lipid storage). As previously shown for lipid uptake [22], RTCA experiments on 2D cell culture models allowed to detect lipolysis in short-term experiments, that is, few minutes to hours; fluorescent labelling, cell size fractionation and glycerol release could detect significant differences in TG contents after 2 hr (glycerol), 4 h (intensity), 24 h (droplet size changes), and phenotypic effects (requiring transcriptional regulations and protein synthesis) after longer exposure times (24 h–3d) (Figures 2 and 3). The classical methods used to differentiate adipocytes *in vitro* with rosiglitazone and insulin produce only partially differentiated adipocytes, that is, mostly plurilocular and it takes several weeks to obtain mature adipocytes. We found that fatty acid addition such as oleic acid was required to obtain fully differentiated, that is, unilocular adipocytes, after shorter time exposure, that is, 3 days [22]. Moreover, we observed that the population of 3T3L1 adipocytes was highly variable from an experiment to another, as well as in a given experiment where lipid droplets and adiposize may vary from less than 5 µm to up to 50 µm in diameter. Such an heterogeneity was similarly described in adipose tissues [26]. Full cell size analyses of adipocytes in cell cultures may represent cumulative effects of adipogenesis (small droplets and plurilocular cells), lipid accumulation in mature adipocytes and lipolysis. In order to specifically analyse mature adipocytes, cell size analyses were performed using both lipid droplet size (up to 50 µm in diameter for mature adipocytes), the number of droplets per cell (one in mature adipocytes) and the frequency of droplets with size up to 50 µm on the bases of the range of cell size in rat epididymal tissue (Figure 1) as previously shown [7]. Adipogenic 3T3L1 and sub-cloned 3T3L1-MBX cell lines differentiate, partially or fully, respectively. After treatment with fatty acids 3T3-MBX adipocytes present a more homogeneous distribution of droplet sizes than 3T3L1. However, cell size analyses revealed that in cell cultures as well as in rat explants, this increase in size was not observed in the presence of low glucose concentrations. The lipogenic properties of glucose are well documented. In addition to represent a substrate for *de novo* TG synthesis through glucose transporter Glut4, which expression increases during adipogenesis [27], a burst of high glucose concentration is required during at least the first 3 days of differentiation to induce Glut4 through Glut1-mediated glucose uptake [28]. *Glut1* expression is down regulated during adipogenesis, inversely *Glut4* increases, thus glucose uptake then is dependent on insulin activation of Glut4 [29] (Figure 10(a)). Realtime experiments revealed that 3T3L1 adipocytes treated with HG in short term experiments (2–4 hr) contained less TG than in LG (Figure 3) and release glycerol (Figure 3(a)), thus suggesting induction of lipolysis, named basal lipolysis *versus* catecholamine-induced lipolysis. Similar effects were obtained by AMPK activation, as well as by inhibition of ATGL or FABP4. AMPK regulates basal lipolysis through inhibition of HSL/ATGL pathway [30]. FABP4 secretion is induced by adenylate cyclase-PKA and guanylyl cyclase-PKG dependent lipolytic mechanisms [31,32]. In adipocytes, AMPK is active even in high glucose concentrations and its antilipolytic activity has been previously depicted [30,33,34]. AMPK activity is reduced by increasing ATP/AMP ratio [35], thus possibly by glucose uptake [36], leading to increased basal lipolysis (Figure 10(a)). Our results suggest that glucose itself may be sufficient to induce the lipolytic pathway (Figure 3). Moreover, glucose regulates the transcription of several genes commonly regulated by insulin and fatty acids in adipose tissues, several of them are involved in the regulation of fat mass and involved in obesity (Figure 9(a)). In another way, glucose regulates numerous genes specifically over-represented in adipose tissues through common signalling pathways, noticeably CEBPA, (although regulatable by fatty acids, LKB1, PPARs) and ChREBP. ChrEBP has been previously found to enhance PPARg adipogenic programme [37] during *de novo* lipid synthesis, resulting in a higher increase of lipid storage to synthesis than release by lipolysis observed during adipogenesis (Figure S1). Under our experimental conditions, the inhibition of lipolysis by insulin detected in high *versus* low glucose culture media worked perfectly and resulted in adipose cell size increase (Figure 5, S2 Videos). Results show that the both ATGL inhibition nor insulin could counteract oleic acid induction of size increase. It is important to notice that insulin was efficient in a dose-dependent manner (Figures (2e) and (2f)) and no insulin resistance was observed in the range of concentrations used (OA 10 µM or less) since insulin exerted its protective effect in rat adipose tissues, 3T3L1 and 3T3-MBX cell lines as well as on the primary cell culture of human adipocytes (anti-lipolytic effect, Figure 4, and gene transcription analyses, Figure S4). Indeed, insulin treatment in presence of high glucose was related to an increase transcriptional level of adipogenic markers CIDEC, FABP4 and FAT/CD36 after 3 days in 3T3-MBX (Figure 7) or 48 h in human adipocytes (Figure S5). Since we have previously published that insulin did not exert a significant effect in 24 h on *CIDEC, FABP4* and *FAT/CD36* gene transcription although they were induced by oleic acid in 3T3L1 adipocytes [22] the pro-adipogenic activity of insulin is more expected to come from *novo fatty* acid synthesis induction rather than direct transcriptional regulation of adipogenic markers. Although OA had similar effects on HG *versus* HG plus insulin on adipogenic markers after 48 h in human or 3 days in 3T3L1, it can directly activate their gene transcription [22]. Only *CIDEC* gene transcription was reduced by OA in HG plus insulin, but not in HG. A recent study showed that in presence of high glucose, insulin limited the synthesis of glycerol-3P from glucose and its incorporation into acyl-glycerides [38,39].

(A) Antilipolytic activity of insulin is a short-term event against glucose induced ATGL reactivation, through reduction of AMPK activity, together with *de novo* fatty acid synthesis from glucose, thus promotes lipid storage during adipogenesis and avoid adiposize reduction of mature adipocytes. (B) High fatty acid uptake (oleic acid) requires high glucose concentration for storage as triglycerides, together with lipolysis inhibition through AMPK activation as short term events, but requires, as long term event, protein synthesis for lipid droplet size increase, through FAT/CD36 mediated PPARs transcriptional activation. (C) Although its antilipolytic activity described in (A) is not altered, insulin may prevent adiposize increase due to high lipid uptake through AMPK inhibition, thus reactivation of ATGL, consequent release of fatty acids activators of PPARA and its inhibitory effect on the transcription of *CICEC* and *G0S2*, two major genes involved in lipid droplet synthesis.

An interesting observation is that in the presence of oleic acid, insulin decreases lipid content and the frequency of large adipocytes. This was observed not only on 2D cell cultures but also on adipose tissue explants, and confirmed by its repressive activity on oleic acid induced gene transcription in 3T3L1, 3T3-MBX and human adipocytes. It is well known that insulin exerts an anti-lipolytic effect under both basal conditions and catecholamine induced lipolysis [40]. The binding of insulin to its membrane receptor activates insulin receptor substrates 1 and 2, then phosphatidyl inositol 3 kinase complex (PI3 Kinase) (Figure 10(a)).

Subsequently, the PKB/Akt pathway is stimulated, resulting in the activation of phosphodiesterase 3B which catalyses the degradation of cyclicAMP, thus reduces AMPK activity. Furthermore, it is also known that AMPK inhibits HSL activation by its phosphorylation at S565, thus prevents the phosphorylation by Akt at S563 or S660, resulting in the inhibition of lipolysis [40]. AMPK can be phosphorylated at S485 in a PKB-dependent manner in response to insulin, and this was associated with a reduction of AMPK activity [41] (Figure 10(a)). Moreover, fatty acids activate AMPK which itself increases glucose uptake and thus its availability for TG synthesis [41–43] (Figure 10(b)). Activation of LKB1/AMPK pathway as well as inhibition of PPARs were also found to counteract the protective effect of insulin in 3T3L1 adipocytes (Table 1). Thus, AMPK inhibition by insulin may consequently increase lipolysis through reactivation of ATGL (Figure 10(c)). However, since inhibition of ATGL was not sufficient to reverse insulin effect (Table 1), the regulation of lipolysis was not the major regulator of adiposize for adipocytes treated with excess of fatty acid. The protective effect of insulin was found to be related to inhibition of the transcriptional activity of oleate on genes required for lipid droplet formation [44–47] but also of its own receptor (*INSR*) (Figure 6). Several transcriptional pathways regulated by insulin are possibly involved. Although PPARG is the major transcription factor regulating adipogenesis, we found that although its transcription was not affected in 3T3-L1 adipocytes, it was repressed by oleic acid in both 3T3-MBX and human adipocytes (Figure 7, Figure S5) and inhibition of its activity affected droplet size distribution in 3T3L1 but not in 3T3-MBX adipocytes (Table 1). This result is in accordance with Sauma and coll. [48] which showed a reduction of PPARG contents in mature primary human visceral adipocytes.

Insulin counteracts the transcriptional activation by OA of proteins involved in lipid droplet formation, such as *CIDEC and G0S2*. In a previous study we found similar results, and *CIDEC* was induced by oleic acid through FAT/CD36 signalling[22]. CIDEC itself regulates through transcriptional regulations many metabolic pathways, including reduction of FA beta oxidation and glycolysis [49], promotes lipid droplet clustering and then fusion to regulate triglyceride accumulation, inhibits *ATGL* gene transcription, which stability is also regulated by AMPK [50–54]. G0S2 is also an inhibitor of ATGL, thus is antilipolytic, and is inversely regulated at the transcriptional level by PPARA (inhibition) and PPARG (activation) [55–57]. Insulin is able to increase the transactivation capacity of PPARA by phosphorylation [58]. PPARA/PGC1A complex, which regulates mitochondrial biogenesis and oxidative phosphorylation, is itself activated by ligands generated by ATGL [59]. In adipocyte-specific ATGL knockout mice, PPARA target genes are also down-regulated [60]. In our study, inhibition of PPARA activity was found to counteract the protective effect of insulin against oleic acid induced increase of adiposize (Table 1). Taken together these results suggest that insulin counteracts OA-induced gene transcription through regulation of PPARA transcriptional activity, possibly by regulating AMPK/ATGL pathway (Figure 10(c)).

Thus our observations suggest that insulin prevents OA-induced size increase, associated with a transcriptional repression of PPARG gene, through PPARA mediated transcriptional regulation of genes involved in lipid storage, such as *CIDEC* and *G0S2*.

In conclusion, our results suggest that in adipocytes insulin signalling proceeds differentially in the regulation of adipogenesis and lipogenesis *versus* concomitant fatty acid uptake. Insulin promotes both adipogenesis (adipogenic gene transcription) and lipogenesis (TG accumulation from *de novo* synthesis by Glut4-induced glucose uptake and inhibition of lipolysis). OA promotes both adipogenesis by induction of adipogenic genes (but repression of PPARG) and TG uptake. Insulin counteracts OA-induced increase of adipocyte size at least through regulation of several adipogenic genes related to PPARA activity. These results suggest a pivotal role of PPARA in the regulation of adiposize, which should be further explored.

Obesity induces insulin resistance in adipose tissue [61]. At the cell level, the consequence of insulin resistance means the loss of the antilipolytic effect of insulin. Insulin is not anymore able to prevent adipocyte hypertrophy. Generally, insulin resistance is seen as a systemic phenomenon occurring at the tissue level. It may very well appear progressively reaching isolated cells inside an adipose lobule and then propagates from cell to cell to finally impact the majority of cells inside a fat lobule. This could explain the macrophage crown observed within adipose tissue of obese patient, in which macrophages target one adipose cell, for so far, unknown reason. This could also explain adipose cell size heterogeneity. Indeed, the repartition of adipose cells according to their size inside one adipose tissue depot, shows that few cells may become very large. In a previous study we showed that the 10% larger cells were positively associated with concentrations of plasma triglycerides and HOMAir index [62] (10.1530/EJE-15-0822).

## Material & methods

### 1- Cell and explant cultures and treatments

Explants were obtained from fresh surgery of rat epididymal adipose tissues maintained in Hepes media then cut into approximately 1 mm^3^ fragments.

Human ASCs were obtained from an anonymous donor (non-diabetic female 38 years-old, 1.73 m height, 98 kg, BMI 32.1) according to French regulation after declaration to research ministry (DC n°2,008,162). 3T3L1 and 3T3-MBX sub-clone cell lines (Sigma-Aldrich, Saint-Quentin-en-Yvelines, France) and human ASCs were grown in Dulbecco’s Modified Eagle’s Medium (DMEM) with glucose 4.5 g/L (HG), foetal calf serum (FCS) 10% containing antibiotics (Thermo Fisher Scientific, Illkirch, France) and basic Fibroblast Growth Factor (bFGF 20 ng/mL, Sigma Aldrich) for ASCs. Adipogenesis was induced and cells analysed as described in Berger and Coll. [22]. The list of drugs is reported in Table S4 With exception to free glycerol analyses, all experiments were performed using stabilized human insulin Actrapid (0.05 U/mL). Briefly, cells were plated at 5000 cells/cm^2^ using Scepter counter® (Millipore, Burlington, USA) in either 96-wells E-plates or 96-wells plates for Real Time Cell Analysis (RTCA) on xCelligence system® (ACEA Biosciences Inc., Agilent, Santa Cruz, USA), cell size imaging or in 12-well plates for Multisizer® (Beckman Coulter, Villepin, France) cell size fractionation and for conditioned media analyses, 6-wells plates for total RNA extractions, and grown in culture media until 90% confluency. Differentiation was induced in growth culture media containing antibiotics and differentiation cocktails 1 (rosiglitazone 20 µM, insulin 0.05 U/mL, IBMX 0.25 mM, dexamethasone 0.25 mM, Sigma Aldrich) during 24 h then differentiation cocktail 2 (rosiglitazon 20 µM, insulin 0.05 U/mL) 24–48 h then insulin 0.05 U/mL until time of experiment [22].

Oleic acid was prepared as a stock solution 400 µM complexed to lipid-free bovine serum albumin (BSA, Sigma Aldrich) 10% by incubation at 42°C during 2 hours then filtered and stored at −20°C until use. At time of experiments it was diluted at 10 µM in 1% BSA or human lipid-free albumin for human cells (Sigma Aldrich), in either low or high glucose medias (LG, 1 g/L and HG, 4.5 g/L, respectively) with or without FCS (10%) then incubated at 42°C during 2 hours.

### 2- Cell culture analyses

The methods were previously described [22]. Mouse adipose cell lines 3T3L1 and sub-clone 3T3L1-MBX were respectively used in order to analyse either unilocular adipocytes (partial differentiation) or signalling pathways (90–100% differentiated). Briefly, cell cultures were analysed in realtime experiments onto xCELLigence system (ACEA, Agilent Technologies, Les Ulis, France) and Cytation 3 imaging platform (Biotek Instruments, Winooski, USA) then directly or after cell dissociation using trypsin 0.05%. Cells were fixed with formalin 3% (Sigma Aldrich) then labelled with either AdipoRed (Lonza, Ozyme, Montigny Le Bretonneux, France) or after permeabilization with triton 0.1% for Hoechst 33,258 (Sigma Aldrich). They were analysed by either fluorescence intensity quantification, or cell count for each wavelength after normalization to controls on images obtained at objective x4 in 6 to 12 replicates. In short term experiments (3–4 h), TG accumulation was calculated as the ratio of AdipoRed fluorescence intensity increase after 30 min from time of AdipoRed loading (corrected from blank without cells) in order to avoid well differentiating state differences. In longer time experiments (24 h–3 days), cells were fixed before labelling and analysed for droplet volume and droplet number per cell (i.e. droplet number normalized to Hoechst counts) in x4 images using automated image acquisition and analysis software Gen5 2.08 from Biotek. TG contents (volume) per cell were obtained as the mean number of droplet per cell x mean droplet volume. In each experiment, imaging parameters (i.e. led intensity, camera gain, threshold) were optimized and applied to each sample. The exact number of droplets could be underestimated in experiments using OA treatment due to limits of high Adipored intensities, in comparison to experiments on small sized adipocytes. Therefore, in experiments using inhibitors, fold changes to corresponding controls were preferred to absolute values. In cell cultures, mature adipocytes were considered for diameters up to 50 µm and were analysed according to mean droplet volume and frequency.

Explant images obtained on Cytation 3 platform (objective x 4) were analysed using cell surface analysis with ImageJ software. Cell sorting and analyses were performed using Novocyte cytometer (ACEA, Agilent) through analysis of 10000 cells per fraction in triplicates. Cell size fractionation was performed in triplicates using a Multisizer cell counter with 400 µm aperture on 10000 cells per sample. Glycerol release in culture media was quantified using ‘Adipolysis Assay’ kit and the control isoproterenol included in the kit (Sigma Aldrich).

### 3- Immunocytochemistry

Cells were labelled with two mouse anti-FAT/CD36 antibodies directed to the extracellular domain, coupled to either phycoerythrin (PE) for cell sorting, or APC (Ozyme) for fluorescence quantification on x4 images, without permeabilization in order to detect only extracellular FAT/CD36 addressing. After dissociation for cell sorting analyses, or fixation with paraformaldehyde 3%, cells were blocked with 5% FCS, 1% BSA in PBS, incubated with anti FAT/CD36 antibodies (2.5 µg/mL) in blocking solution, then rinsed three times in PBS before analyses. Times of treatment were, respectively for either cell sorting and image quantification, 1 or 2 hours at room temperature in blocking solution, then 2 hours at room temperature or overnight at 4°C, respectively. Fluorescence image quantifications (Delta APC- blank without antibody obtained with identical acquisition parameters) were normalized to nuclei Hoechst counts, that is, cell number. Tests were performed in 8 replicates of 96-wells plates.

### 4- mRNA quantifications

Adipocytes were differentiated in 6-well plates, treated then dissociated in Trizol Reagent (Sigma Aldrich). Total mRNA purification, reverse transcription and quantitative real-time polymerase chain reaction (SYBRGreen kit, Roche Diagnosis) were performed as previously described [22]. Relative concentrations were deduced from Delta Ct, performed in quadruplicates and normalized to hypoxanthine guanine phosphoribosyl transferase (*HPRT*) standard gene. Gene names and ID, Primers and hybridization temperatures are listed in Table. S9.

### 5- Bioinformatics gene dataset analyses

The method for human gene dataset analyses was previously described [17,19–21]. The list of datasets implemented are reported in Table.S7A Briefly, human gene datasets were retrieved from either Gene Expression Omnibus (GEO) datasets or published experiments leading to identification of lists of genes regulatable according to adipose phenotype (ASCs, differentiating adipocytes dA, isolated adipocytes or tissues, AT), extracellular signals, intracellular signalling pathways or transcription factors human gene datasets. The specific enrichment in target genes in comparison to the genome revealed enrichment in signalling pathways, for example between human AT *versus* ASCs. Signalling pathways enriched in fatty, insulin, LKB1/AMP and JAK2 human gene datasets are reported in S7B-D, respectively; those of glucose have been previously published [21]. Significant enrichments were considered for z-test confidence levels >95%.

### 6- Statistics

Experimental data were analysed on representative experiments on three (6 and 12-wells plates) to 6–10 biological replicates (96- and E96-wells plates) at least in three independent experiments, except for dose-dependent response analyses. Statistical analyses were performed using R studio software 4.2.0 (2022–04-2022 ucrt, The Foundation for Statistical Computing platform: x86_64-w64-mingw32/x64). The normality of data distributions were tested (Shapiro), then Students, Wilcoxon (1 condition) or Fisher, Anova and Tukey tests were applied for multiple conditions; significantly different values p < 0.05 are reported as different letters.

## Supplementary Material

Supplemental Material

## Data Availability

Data available within the article or its supplementary materials.
