# Supplementary material for: Insulin prevents fatty acid induced increase of adipocyte size
Source: Adipocyte. 2022 Aug 22;11(1):510–28. doi: 10.1080/21623945.2022.2107784 (PMC9450899; doi:10.1080/21623945.2022.2107784)
Supplement: Supplemental Material [file KADI_A_2107784_SM3247.zip › SUPPLEMENTARY/Suppl_Information_new (3).pdf]

S1 Figure: TG content in 3T3-L1 adipocytes increases with glucose and insulin after 24 h through *de novo* fatty acid synthesis.

TG accumulation in adipocytes was analyzed by image quantification as mean TG content (i.e. volume) per cell, as a result of the product of droplet number per cell and their volume. Cells were treated in culture media with either 1 g/L (LG) or high glucose (HG, 4.5 g/L) and/or insulin (INS 0.05 U/mL). Droplet size and number were counted on x4 images and normalized to nuclei number (Hoechst counts). Analyses were performed either 24h (A) or 3 days (B-C) after treatment (T0). Data are presented as mean values +/-SEM of TG volumes (i.e. droplet volume x number of droplet per cell) in A and B (4 images of 8 replicates in a representative experiment) with significativity represented by letters in Anova variance tests or Student t-test p-values $p<0.05$ for pair comparisons. During differentiation, adipocytes (dA) present several droplets which increase in size and in frequency, resulting in increased TG contents (1A) but do not fuse into a unique, large droplet like mature adipocytes. In partially differentiated adipocytes, glucose 1 g/L (LG) was sufficient to increase TG contents (24 h) with increased effects of 4,5 g/L (HG) after 3 days and additional effect of insulin (1B), both in droplet number and size (1C).

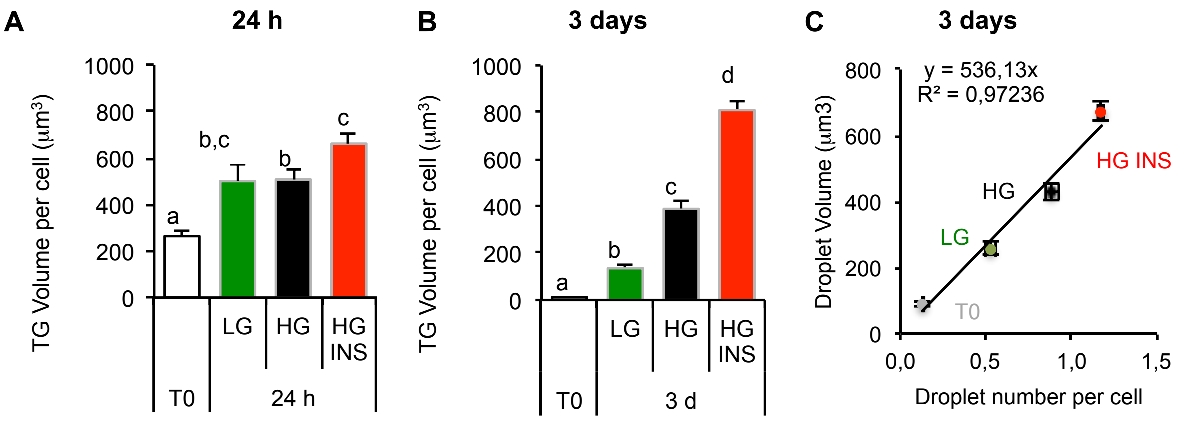

S2 2 Videos

S2 videos: Realtime imaging of oleic acid uptake and basal lipolysis in 3T3MB dA (x20 on Cytation3)
A- 3T3MB adipocytes treated during 4 days in HG culture media with OA 10 µM.
1 image /30 min. *(*reconstitution 1 image/2s*)*.

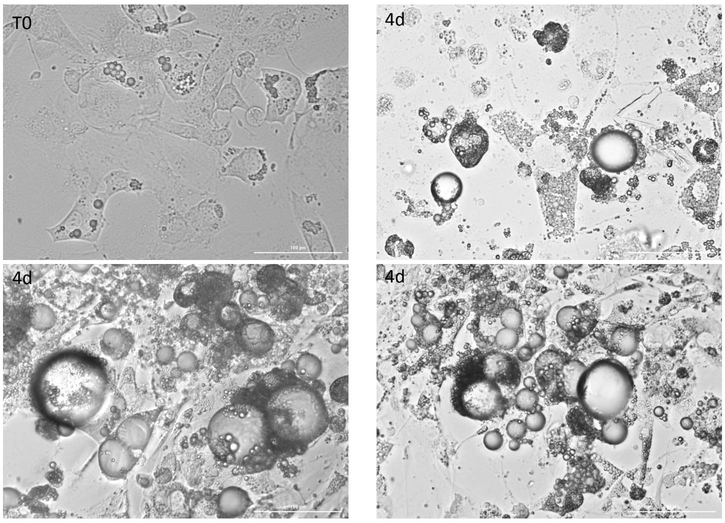

HG after OA 24h.mp4

HG after OA 24h2.mp4

B- Realtime monitoring of basal lipolysis in 3T3MB adipocytes pre-treated with 0A 10 µM during 24h then in either LG, HG or HG +INS culture media (24 h). 1 image/30 min. (reconstitution 1 image/2s).

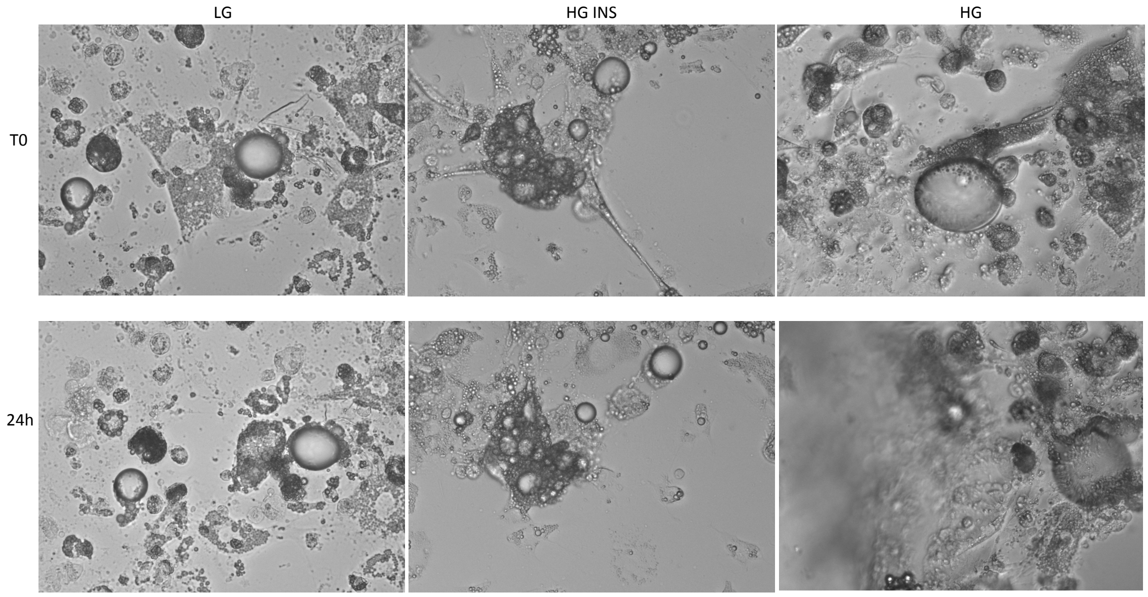

LG after OA 24h.mp4
HG INS after OA 24h.mp4
HG after OA 24h.mp4
HG after OA 24h2.mp4

S3 Table. TG content analysis by analysis of AdipoRed fluorescence intensity in 3T3L1 adipocytes treated during 2 hours.

Data are presented as mean fold change of AdipoRed fluorescence intensity to that of corresponding control media (DMEM HG 4.5 g/L or DMEM HG INS 0.05 U/mL, no significant effect of inhibitor solubilisation media, not shown) with significant Student's t-test p-values<0.05, n=8 replicates). The results were retrieved from independent experiments for each treatment.

Drug	HG	t-test	HG INS	t-test
Forskolin	1,00 ± 0.05		1.19 ± 0.09	
GSK69 (I)	0.68 ± 0.06		0.95 ± 0.06	
**AICAR (A)**	**2.16 ± 0.19**	**0.014**	1.04 ± 0.15	
WL4003 20 ng/mL (NUAK1) (I)	1.27 ± 0.08	**0.0002**	0.98 ± 0.02	
WL4003 100 ng/mL (NUAK2) (I)	1.11 ± 0.08		1.07 ± 0.04	
**ATGListatin (I)**	**2.41 ± 0.12**	**0.014**	1.02 ± 0.11	
KN93 (I)	1.08 ± 0.03		1.08 ± 0.03	
**FABP4i (I)**	**1.98 ± 0.24**		0.93 ± 0.13	
GTPg (A)	1.06 ± 0.01		0.96 ± 0.01	
GSK137647 (A)	1.02 ± 0.02		0.97 ± 0.01	
Pertussis toxin	0.95 ± 0.04		0.97 ± 0.01	
**Low Glucose**	**3.07 ± 0.22**	**0.0046**		
GSH (I)	1.03 ± 0.05		1.00 ± 0.05	
**Insulin**	**1.80 ± 0.073**	**0.0002**		
**Metformin (A)**	0.89 ± 0.06		**0.77 ± 0.03**	**0.0121**
**Oligomycin (A)**	0.87 ± 0.10		**0.75 ± 0.02**	**0.0031**
Compound C (I)	0.78 ± 0.07		0.78 ± 0.04	
Butein (I)	1.02 ± 0.07		1.01 ± 0.04	
Tyrophostin 490 (I)	1.13 ± 0.04		1.05 ± 0.07	
OA 5 µM	**1.56 ± 0.10**	**0.0187**	0.56 ± 0.05	**0.0008**
OA 10 µM	**1.43 ± 0.03**	**0.0142**	1.25 ± 0.02	
**LY94002 (I)**	**0.68 ± 0.04**	**0.0002**	**0.69 ± 0.04**	**0.0001**
U73122 (I)	1.21 ± 0.07		0.70 ± 0.06	
GW6471	1.01 ± 0.03		1.1 ± 0.11	
GW9662	0.95 ± 0.05		1.04 ± 0.04	

S4 Figure: qRTPCR analysis of adipogenic genes quantified in both 3T3L1 adipocytes *versus* fibroblasts and Human primary adipocytes *versus* adipose stem cells (fibroblasts from de same donnor). Data are presented as mean fold change mRNAs (normalized to HPRT gene) in adipocytes *versus* fibroblasts (n=3 biological replicates, 4 technical replicates) with significant Student t-test p-values (p<0.05).

Human adipocytes				
**Gene symbol**	**Fold change dA/ASCs**			p-value
Up-regulated				
CEBPA	3,28	±	0,05	0,0002
CIDEC	3,05	±	0,13	0,0008
FABP4	induced			0,0005
FAT/CD36	15,48	±	0,14	5,4E-06
PGC1A	2,62	±	0,01	0,0027
PPARG2	1,83	±	0,10	0,0039
TSPAN8	6,33	±	0,17	0,0110
Down-regulated				
CCND1	0,47	±	0,01	2,7E-05
GPR120	0,61	±	0,03	0,0021
REDD1	0,38	±	0,01	0,0002
Not regulated				
CIDEA				

3T3L1 adipocytes				
**Gene symbol**	**Fold change dA/fibroblasts**			p-value
Up-regulated				
CEBPA	16,49	±	0,49	5,5E-06
CIDEA	52,77	±	0,14	7,8E-08
CIDEC	53,47	±	0,54	6,4E-08
FABP4	induced			3,4E-03
G0S2	8,34	±	0,13	9,8E-07
PPARG	3,49	±	0,11	2,2E-03
Down-regulated				
FN1	0,10	±	0,00	1,9E-06
INSR	0,33	±	0,01	7,6E-05
MMP9	0,04	±	0,00	5,5E-08
Not regulated				
PTGS2				

S5 Figure: Gene transcription analysis by qRT PCR *in vitro* differentiated human adipocytes treated during 48 h with either insulin (INS) 0.05 U/mL or several doses of oleic acid (OA, μM) in culture media (4.5 g/L glucose) and corresponding stem cells (ASCs) (prepared in culture media containing bFGF). Data are presented as mRNA normalized to that of HPRT gene) (n=3 biological replicates, 3 technical replicates). Different letters represent significant differences (Anova then Tukey tests for p-values p<0.05).

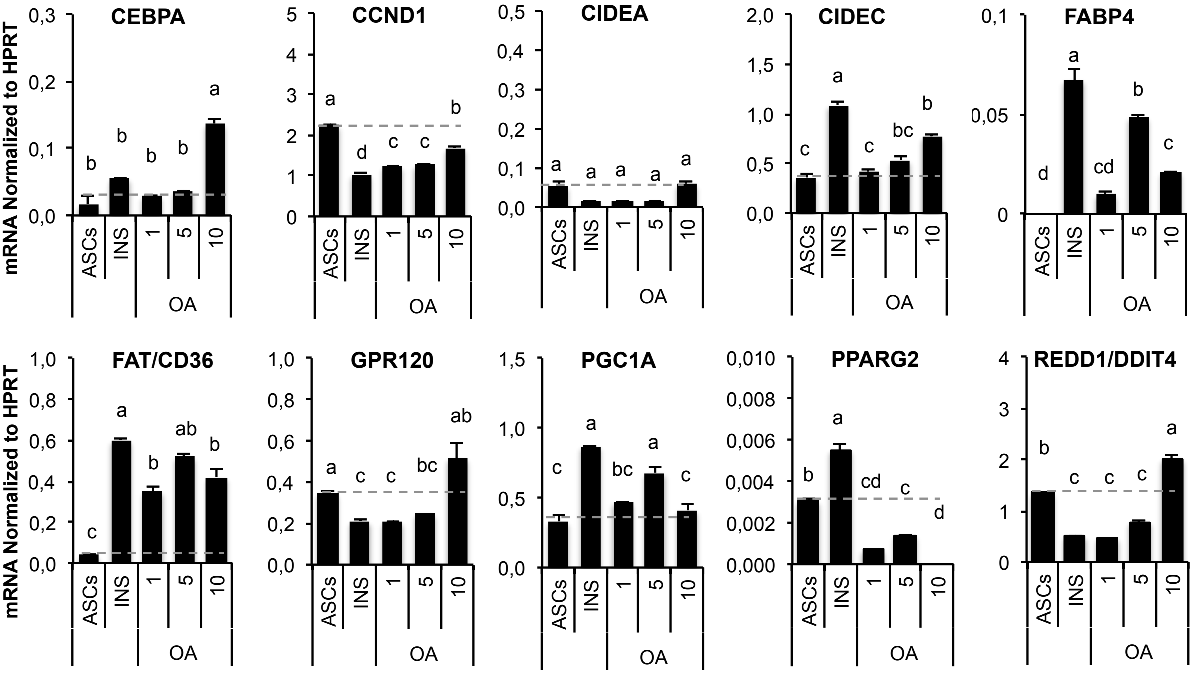

S7 Figures: Gene dataset analyses of signaling pathways enrichment in human adipose tissue. Only significantly over-represented pathways are reported and compared to genome, on the basis of z-test confidence level > 95%.

Figure 7A: Gene dataset list implemented from Berger et al, 2015 [22] in red. Human gene datasets retrieved from either Pubmed (PMID) or Gene Expression Omnibus (GEO) identifier and their frequency in genome (adjusted to the number of genes with at least one regulator i.e. 12035 genes).

**Pathway**	**n**	**%**	**PMID**	**Reference or GEO dataset**
		**Stimulus (49 pathways)**		
Epidermal Growth Factor (EGF)	585	4,5		GPL4044
FABP4	2406	15,1	27936164	Yamamoto et al 2018
FABP5	1585	10,0		
Macrophage on preA	472	3,6	19585142	O'Hare et al, 2009
Macrophage on TA	325	2,5	19585142	O'Hare et al, 2009
		**Intracellular (73 pathways)**		
HER2	377	2,664	17660348	Gometz et al, 2007
Lipid storage in adipocytes	333	2,8	22384002	Söhle et al, 2012
		**Transcription (69 pathways)**		
Acetylation adipose	6945	41,1	21655096	Lo et al, 2011
CEBPA acetylation adipose tissue	4262	30,12	21655096	Lo et al, 2011
cAMP response element-modulator (CREM)	336	2,4	22121215	Hurley et al, 2012
E2F4 acetylated adipose tissue	4621	32,66	21655096	Lo et al, 2011
ERRA	557	4,9	18974123	Stein et al, 2008
ESR1, ESR2	845	7,4	18974123	Stein et al, 2008
Peroxisome proliferator-activated receptor gamma coactivator 1-alpha (PPARGC1A)	1084	9,5	18974123	Stein et al, 2008

S7B Figure: Signaling pathway enrichment in human adipose tissue phenotype.

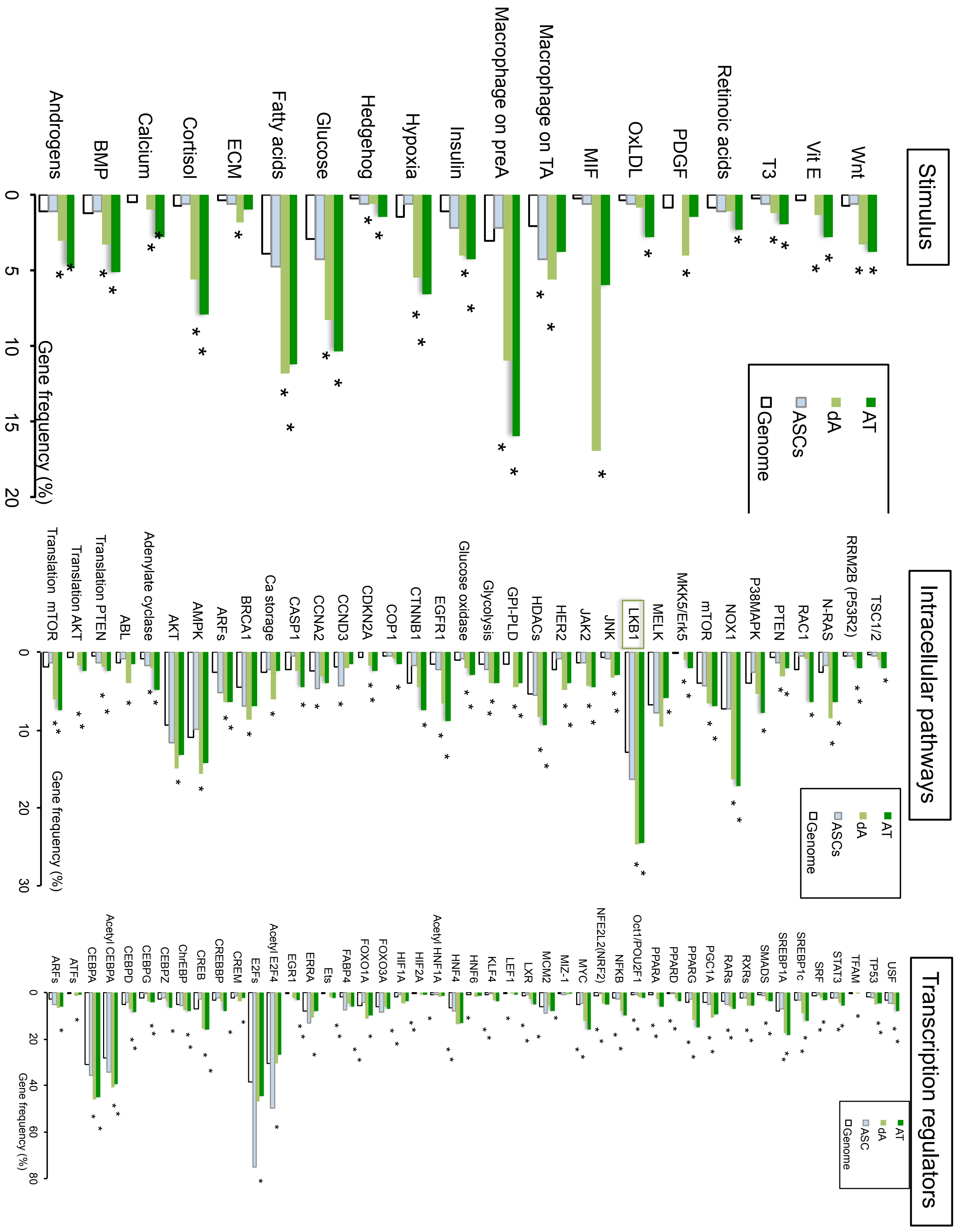

S7C Figure: Signaling pathways enrichment in human insulin gene dataset.

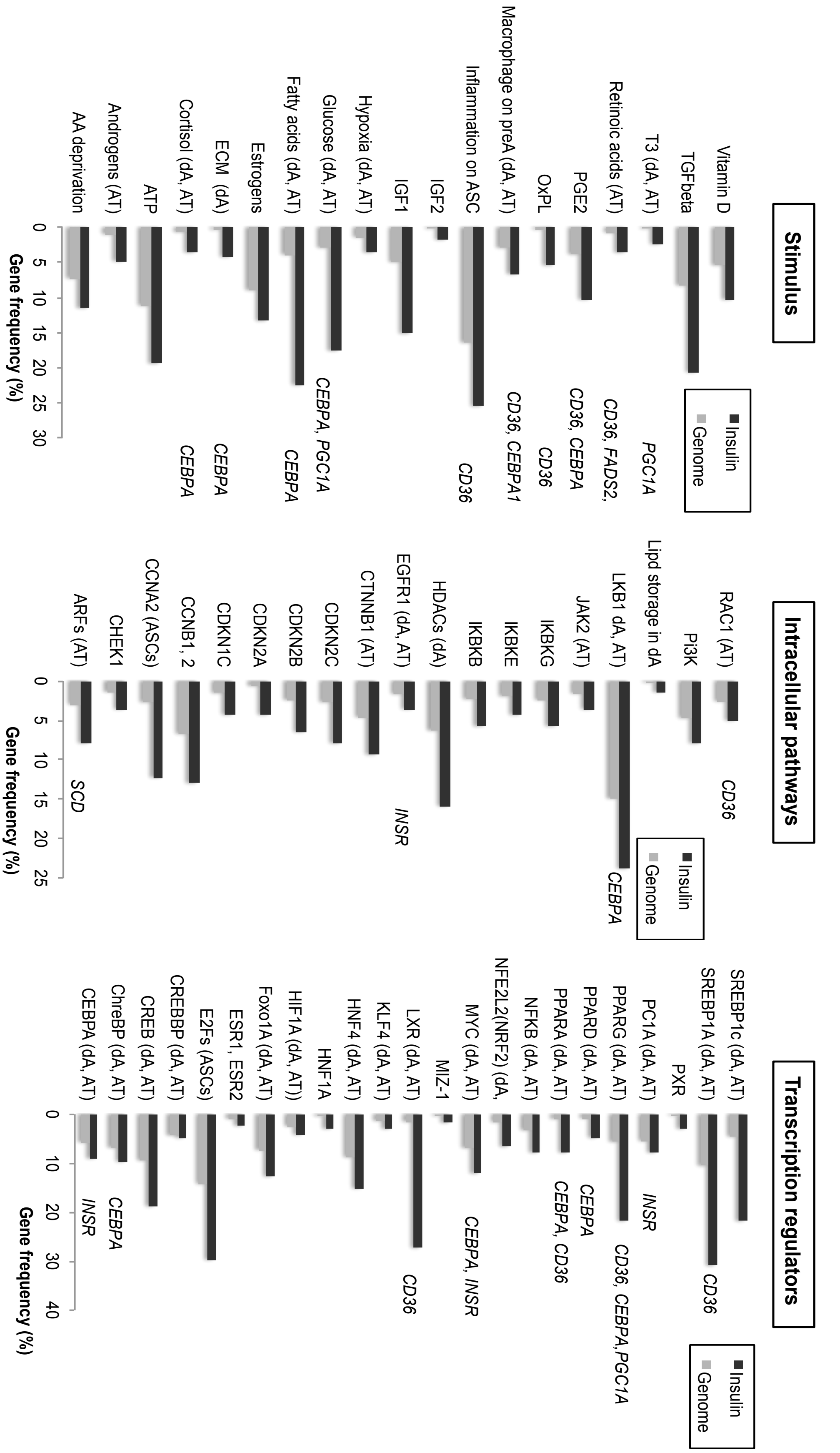

S7D Figure: Signaling pathway enrichment in fatty acid gene dataset

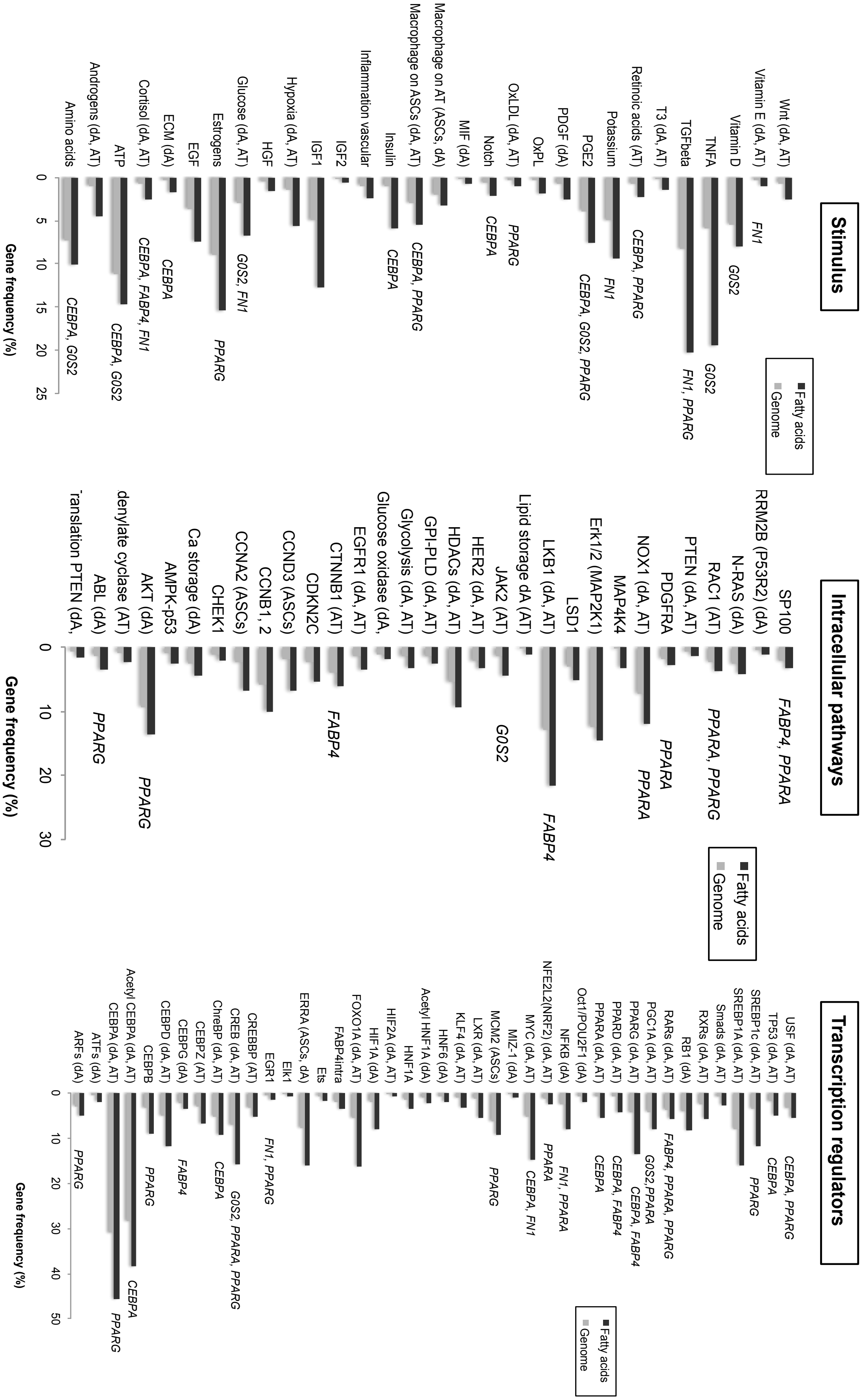

S7E Figure: Transcription regulator pathways enriched in LKB1/AMPK target gene dataset.

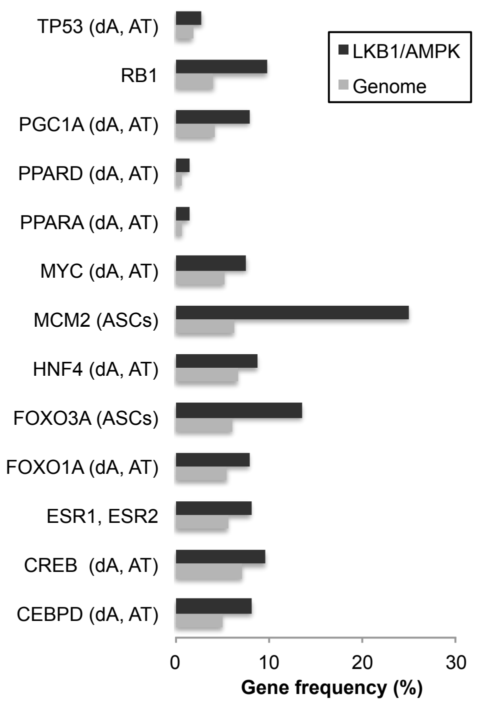

S7F Figure: Transcription regulator pathways enriched in JAK2 target gene dataset.

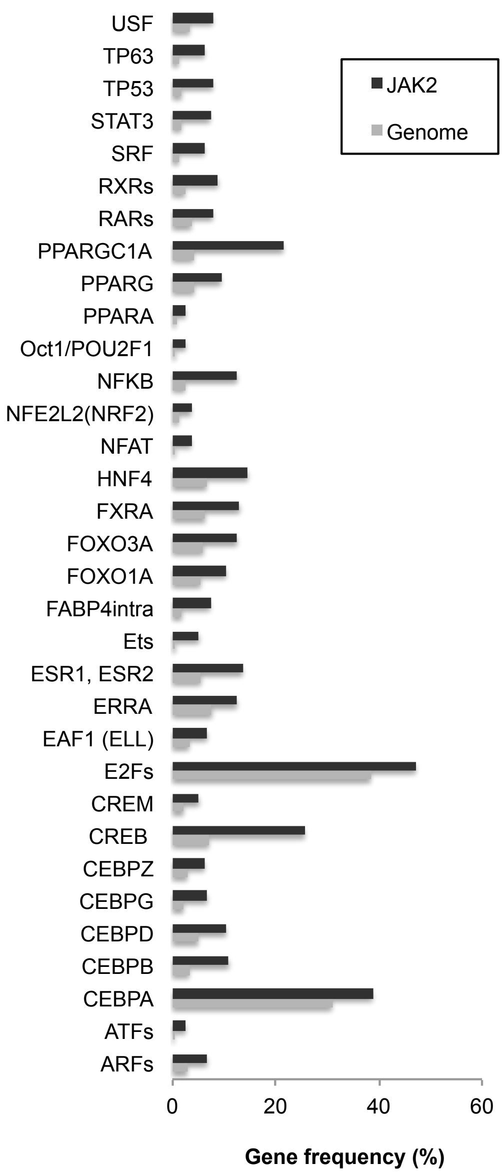

S8 Table: Table List of drugs used at optimized concentrations and corresponding pathways they regulate as activators or inhibitors.

Drug	Purchased by	Properties	Reference (PMID or IC50)	Concentration
**AGONISTS**				
AICAR	Sigma Aldrich	LKB1/AMPK	16901342	1 mM
bFGF (basic fibroblast Growth factor)	Sigma Aldrich		20042795	10 ng/mL
Forskolin	Sigma Aldrich	Adenylate cyclase	1314877	20 nM
L-glutathion reduced (GSH)	Sigma Aldrich		IC50	10 µM
GSK13764747	Sigma Aldrich	FFA4/GPR120	IC50	20 ng/mL
GTPg(S Guanosine 5'-O-(3-Thiotriphosphate*TETR)	Sigma Aldrich	Glut4	9582374	10 µM
Insulin (human)	Actrapid		25595190	0.05 mU/ml
Insulin (recombinant) human	Sigma Aldrich			0.05 U/mL
Isoproterenol	Sigma Aldrich	Beta adrenergic receptor/Lipolysis	Adipolysis kit	100 nM
Metformin (1.1 Dimethylbiguanidine hydrochloride)	Sigma Aldrich	LKB1/AMPK	16901933	1 mM
Oligomycin	Sigma Aldrich	AMPK	IC50	2 µM
Oleic acid	Sigma Aldrich		26257990	10 µM
Rosi(glitazone)	Sigma Aldrich	PPARG	25595190	20 µM
**INHIBITORS**				
AP5258	Clinigenetics	FATCD36	2625799à	10 µM
ATGListatin	Sigma Aldrich	ATGL (lypolysis)	IC50	1 µM
Butein	Sigma Aldrich	Janus kinase 1	9571170	20 µM
CC (Compound C, Dorsomorphin dihydrochloride)	Sigma Aldrich	AMPK	22674626	40 µM
DDA (2,5-dideoxyadoenosine)	Sigma Aldrich	Adenylate cyclase	21444924	1mM
DPH (Dihydroxyacetone phosphate hemimagnesium salt hydrate)	Sigma Aldrich	Glycerol synthesis, lipogenesis	IC50	0,1 mM
DPQ (3,4-Dihydro-5-[4-(1-piperidinyl)butoxyl]-1(2H)-isoquinolinone)	Sigma Aldrich	poly(ADP-ribose) polymerase (PARP)	IC50	1 µM
EX-127	Sigma Aldrich	SIRT1/6 inhibitor	IC50	1 µM
FABP4i(nhibitor)	Sigma Aldrich	Fatty acid binding protein 4	IC50	20 µM
GSK69(0693)	Sigma Aldrich	Glucose synthase kinase 3	28628117	20 nM
GW6471	Sigma Aldrich	PPARA	IC50	10 µM
GW9662	Sigma Aldrich	PPARG	IC50	10 µM
IBMX	Sigma Aldrich	Phosphodiesterase	25595190	1 µM
IRGASAN	Sigma Aldrich	Fatty acid synthasr	IC50	10 µM
KN 93	Sigma Aldrich	Calmodluin kinase II	IC50	10 µM
KT5720	Sigma Aldrich	Protein kinase APMc dependant	IC50	3 µM
LY294002	Cell Signaling	PI3 kinase	22025081	10 µM
P3115	Sigma Aldrich	Protein kinase cGMP	8420972	50 µM
Pertussis Toxin	Sigma Aldrich	Protein Gi/G0	IC50	500 ng/ml
Rapamycin	Cell Signaling	TOR/P70S6Kinase	15870276; 21830446	10nM
SP600125	Sigma Aldrich	Jun-NH2 kinase	14766793	20 µM
Tyr490 (Tyrphostin AG-490)	Sigma Aldrich	Janus kinase 2	18448488	50 µM
U73122	Sigma Aldrich	Phospholipase a-C & phospholipase 2	18629476	5µM
VAS2870	Sigma Aldrich	NADPH oxydase	IC50	25 µM
WZ4003	Euromedex	NUAK1	24171924	20 µM
		NUAK2	24171924	100 µM

S9 Table: KiCqStart® primers from Sigma Aldrich

Gene symbol	Gene name	Gene id	T°
Mouse			
CEBPA	CCAAT/enhancer binding protein (C/EBP), alpha	12606	60°C
CIDEA	cell death-inducing DNA fragmentation factor, alpha subunit-like effector A	12683	60°C
CIDEC	cell death-inducing DFFA-like effector c	14311	60°C
FN1	fibronectin 1	14268	58°C
G0S2	G0/G1 switch gene 2	14373	50°C
INSR	insulin receptor	16337	56°C
MMP9	matrix metallopeptidase 9	17395	56°C
PPARG	peroxisome proliferator activated receptor gamma	19016	59°C
PTGS2	prostaglandin-endoperoxide synthase 2	19225	60°C
Human			
CEBPA	CCAAT/enhancer binding protein (C/EBP), alpha	1050	60°C
CCND1	cyclin D1	595	60°C
CIDEA	cell death-inducing DNA fragmentation factor, alpha subunit-like effector A	1149	60°C
CIDEC	cell death-inducing DFFA-like effector c	63924	60°C
FABP4	fatty acid binding protein 4, adipocyte	2167	60°C
CD36	CD36 molecule (thrombospondin receptor)	948	60°C
GPR120 (FFAR4)	free fatty acid receptor 4	338557	60°C
PGC1A (PPARGC1A)	PPARG coactivator 1 alpha	10891	60°C
REDD1/DDIT4	DNA-damage-inducible transcript 4	54541	60°C

S Table xx Genes commonly regulatable by high glucose, insulin and/or fatty acids in human cells involved in the regulation of fat mass and/or obesity.

Symbol	Name	Gene Id	GO function	Adipose tissue and obesity
**AGPAT2**	1-acylglycerol-3-phosphate O-acyltransferase 2 (lysophosphatidic acid acyltransferase, beta)	10555	**Cellular lipid metabolic process**	Lipodystrophy (Magré et al, 2003, PMID 12765973)
AKR1B1	aldo-keto reductase family 1, member B1 (aldose reductase)	231	Oxidation-reduction process; Response to stress; Fructose biosynthetic process	Increased in obese adipose tissue (Michaud et al, 2014; PMID 24663124)
C1S	complement component 1, s subcomponent	716	Complement activation, classical pathway	Increased expression in obese adipose tissue (Gabrielsson et al, 2003, PMID 12805391)
CTSC	cathepsin C	1075	Proteolysis	Increased in obese adipose tissue (Shea et al, 2009; PMID 19056584)
**FADS2**	fatty acid desaturase 2	9415	**Biosynthesis of unsaturated fatty acids**	Regulation by PUFAs in adipocyte (Ralston et al, 2015, PMID 25755223)
FN1	fibronectin 1	2335	cell adhesion, extracellular matrix	Dysregulated (Berger et Al, 2015)
FTL	ferritin, light polypeptide	2512	Iron ion transport	Modulation by insulino-resistance in adipose tissue (Moreno-Navarrete et al, 2014; PMID 24496804);serum production increased in obesity (Freixenet et al, 2009; PMID 19000667)
**LDLR**	low density lipoprotein receptor	3949	**Lipid metabolic process**	LDLR polymorphism associated with obesity, Mattevi et al, 2000 (PMID 10914685)
PCK1	phosphoenolpyruvate carboxykinase 1 (soluble)	5105	Glycerol biosynthetic process from pyruvate; Response to insulin	Candidate obesuty and diabetes gene (Beale et al, 2004, PMID 15046742)
**PDK4**	pyruvate dehydrogenase kinase, isozyme 4	5166	**Cellular response to fatty acid; Reactive oxygen species metabolic process; Regulation of acetyl-CoA biosynthetic process from pyruvate**	Increased in obese adipose tissue (Shea et al, 2009; PMID 19056584)
**SCD**	stearoyl-CoA desaturase (delta-9-desaturase)	6319	**Fatty acid biosynthetic process**	SCD1 ratio associated with fat mass (Yu Tan et al, 2015; PMID 26679101)
SERPINE1	serpin peptidase inhibitor, clade E (nexin, plasminogen activator inhibitor type 1), member 1	5054	Negative regulation of cell adhesion mediated by integrin; Positive regulation of inflammatory response	Induction by inflammation in adipoisty (Ekström et al, 2023, PMID 22740034)
TM4SF1	TM4SF1 transmembrane 4 L six family member 1	4071		Over-expression in hypertophic adipocytes (Jernas et al, 2008; PMID 16754744) and hyperlipidemia (Miao et al, 2018; PMID 30045016)
**TXNRD1**	thioredoxin reductase 1	7296	**Cellular lipid metabolic process; Response to reactive oxygen species**	
